# Identification of *Hedyotis* *diffusa* Willd-specific mRNA–miRNA–lncRNA network in rheumatoid arthritis based on network pharmacology, bioinformatics analysis, and experimental verification

**DOI:** 10.1038/s41598-024-56880-y

**Published:** 2024-03-15

**Authors:** Jing Jiang, Meng Huang, Si-Si Zhang, Yong-Gang Wu, Xiao-Long Li, Hui Deng, Xin-Yu Qili, Jian-Lin Chen, Yao Meng, Wen-Kui Sun

**Affiliations:** 1 School of Laboratory Medicine, Chengdu Medical College, Chengdu, 610500 Sichuan China; 2 Department of Orthopedics, Xindu District People’s Hospital, Chengdu, 610500 Sichuan China; 3 Department of Orthopedics, Xindu District Hospital of Traditional Chinese Medicine, Chengdu, 610500 Sichuan China; 4 Department of Clinical Laboratory, Sichuan Taikang Hospital, Chengdu, 610213 Sichuan China

**Keywords:** Computational biology and bioinformatics, Molecular medicine, Rheumatology

## Abstract

*Hedyotis* *diffusa* Willd (HDW) possesses heat-clearing, detoxification, anti-cancer, and anti-inflammatory properties. However, its effects on rheumatoid arthritis (RA) remain under-researched. In this study, we identified potential targets of HDW and collected differentially expressed genes of RA from the GEO dataset GSE77298, leading to the construction of a drug-component-target-disease regulatory network. The intersecting genes underwent GO and KEGG analysis. A PPI protein interaction network was established in the STRING database. Through LASSO, RF, and SVM-RFE algorithms, we identified the core gene MMP9. Subsequent analyses, including ROC, GSEA enrichment, and immune cell infiltration, correlated core genes with RA. mRNA–miRNA–lncRNA regulatory networks were predicted using databases like TargetScan, miRTarBase, miRWalk, starBase, lncBase, and the GEO dataset GSE122616. Experimental verification in RA-FLS cells confirmed HDW’s regulatory impact on core genes and their ceRNA expression. We obtained 11 main active ingredients of HDW and 180 corresponding targets, 2150 RA-related genes, and 36 drug-disease intersection targets. The PPI network diagram and three machine learning methods screened to obtain MMP9, and further analysis showed that MMP9 had high diagnostic significance and was significantly correlated with the main infiltrated immune cells, and the molecular docking verification also showed that MMP9 and the main active components of HDW were well combined. Next, we predicted 6 miRNAs and 314 lncRNAs acting on MMP9, and two ceRNA regulatory axes were obtained according to the screening. Cellular assays indicated HDW inhibits RA-FLS cell proliferation and MMP9 protein expression dose-dependently, suggesting HDW might influence RA’s progression by regulating the MMP9/miR-204-5p/MIAT axis. This innovative analytical thinking provides guidance and reference for the future research on the ceRNA mechanism of traditional Chinese medicine in the treatment of RA.

## Introduction

Rheumatoid arthritis (RA) is an autoimmune disease characterized by predominantly chronic polyarticular inflammation. Its pathological features include synovial hyperplasia, angiogenesis, joint degeneration, and loss of joint function^1^. The exact pathogenesis of RA is still unclear, involving the overactivation of immune-infiltrating cells such as T/B cells, macrophages, and dendritic cells, and the abnormal proliferation of fibroblast-like synoviocyte (FLS), which is closely related to environmental and genetic factors^2–5^. Studies have shown that the synovial inflammation of RA is associated with the release of inflammatory factors such as matrix metalloproteinases, tumor necrosis factor α and interleukin-6 after the activation of IL-17, TNF, TLRs, MAPKs, NF-κB and other pathways^6^. In response to such inflammatory reactions, traditional treatment mainly relies on non-steroidal anti-inflammatory drugs, glucocorticoids, and traditional anti-rheumatoid drugs, although widely used, but there are adverse reactions such as slow efficacy, high drug resistance, and gastrointestinal impairment^7^. As traditional Chinese medicine advances and research on RA continues, remedies like Tripterygium wilfordii extract and Baihu Guizhi Decoction have been employed to treat RA^8,9^. These have distinct advantages, such as slowing bone destruction, reducing disease activity, enhancing patient quality of life, and mitigating the toxic side effects seen with drugs like immunosuppressants or hormones. However, they cannot be widely used in clinical practice due to the unclear mechanism of systemic action. Therefore, finding drugs with high safety, fast onset and clear mechanism of action is conducive to the effective treatment and prognosis of RA patients.

Hedyotis diffusa Willd (HDW) is a plant of the Rubiaceae family, containing anthraquinones, flavonoids, polysaccharides and other ingredients. It has a bitter taste and is cold in nature. It is known for its heat-clearing and detoxifying effects, as well as its anti-cancer and anti-inflammatory properties. Moreover, HDW is cost-effective, abundantly available, has minimal toxic side effects, and offers significant therapeutic benefits^10^. In modern research, HDW is mostly used in antibacterial and anti-inflammatory, anti-tumor, and improve immunity, especially the anti-inflammatory effect is more prominent, and it is widely used in nephritis, prostatitis and other diseases^11,12^. At the same time, studies have also found that HDW has shown the effect of inhibiting tumor cell proliferation and inducing apoptosis in vitro experiments such as digestive system tumors, non-small cell lung cancer, colon cancer, ovarian cancer and acute leukemia by regulating the expression of different signaling pathways and target genes^13–16^. Studies have shown that scandoside (SCA), a cyclic ethers terpene component of in HDW, can significantly reduce the expression of NO, PGE2, TNF-α and IL-6 in RAW 264.7 macrophages induced by LPS, and inhibit IκB phosphorylation. These suggested that HDW can inhibit inflammation by regulating the NF-κB pathway^17^. Our previous studies have found that HDW affects REAL, TNF, and IL-6 expression, and regulates the PI3K/AKT signaling pathway to inhibit the proliferation of MH7A in RA-mode cells^18^. However, the anti-RA mechanism of HDW is more complex, and there are still no more definitive results.

In recent years, network pharmacology has become an important tool for studying disease pathogenesis, drug targets, and drug effects^19^. With the development of high-throughput technology, researchers can obtain a large amount of high-dimensional data through gene chips, transcriptome sequencing and other technologies. They can screen out disease-related genes, signaling pathways and functional proteins based on bioinformatics analysis, so as to discover new drug targets and predict drug efficacy, which helps accelerate the speed and efficiency of new drug development^20^. Competing endogenous RNA (ceRNAs) is a new gene expression regulatory mechanism, ceRNAs can competitively bind to microRNAs through microRNA response elements (MREs) to affect gene silencing, and to affect the occurrence and development of the disease. LncRNA S56464.1 has been found to promote the proliferation of FLS through the Wnt signaling pathway as ceRNA of miR-152-3p^21^. LncRNA OIP5-AS1 participates in the initiation and development of RA by regulating the miR-410-3p/Wnt7b signal axis to promote the activation of the Wnt/β-catenin signaling pathway^22^. LncRNA OSER1-AS1 acts as ceRNA in RA-FLS through sponged miR-1298-5p and increased E2F1 expression, thereby affecting RA-FLS proliferation and apoptosis^23^. Based on the role of ceRNA regulatory mechanism in RA, HDW-regulated mRNA–miRNA–lncRNA signaling networks are worth studying.

**Figure 1:**
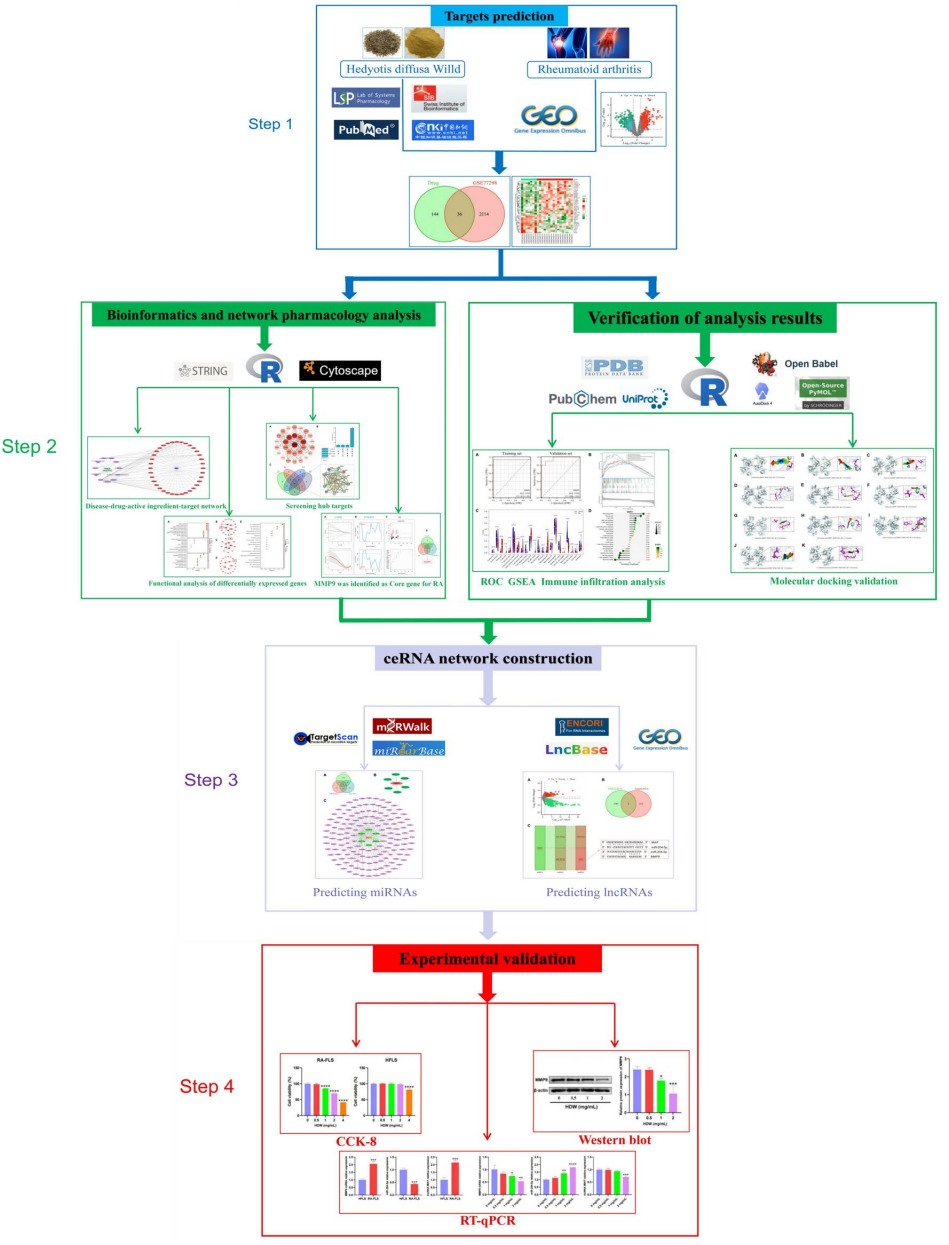
A flow-chart of this study to discusses the active components, potential targets and potential ceRNA mechanism of HDW in the treatment of RA.

Therefore, we intend to use bioinformatics, network pharmacology methods, molecular docking, and cell experiments to investigate the active ingredients, potential targets, and prospective ceRNA mechanisms of HDW in treating RA. The main scheme of this study is presented in Fig. 1. We employed bioinformatics and network pharmacology methods to identify the core targets of HDW against RA. Subsequently, using ROC, GSEA, and immune cell infiltration analysis, we assessed the correlation between these core targets and RA's onset and progression. Based on these core targets, we predicted and screened the mRNA–miRNA–lncRNA axis of HDW. To conclude, we utilized molecular docking and cell experiments to validate the predictions derived from our network pharmacology and bioinformatics assessments.

## Results

### Identify potential targets for RA

**Figure 2:**
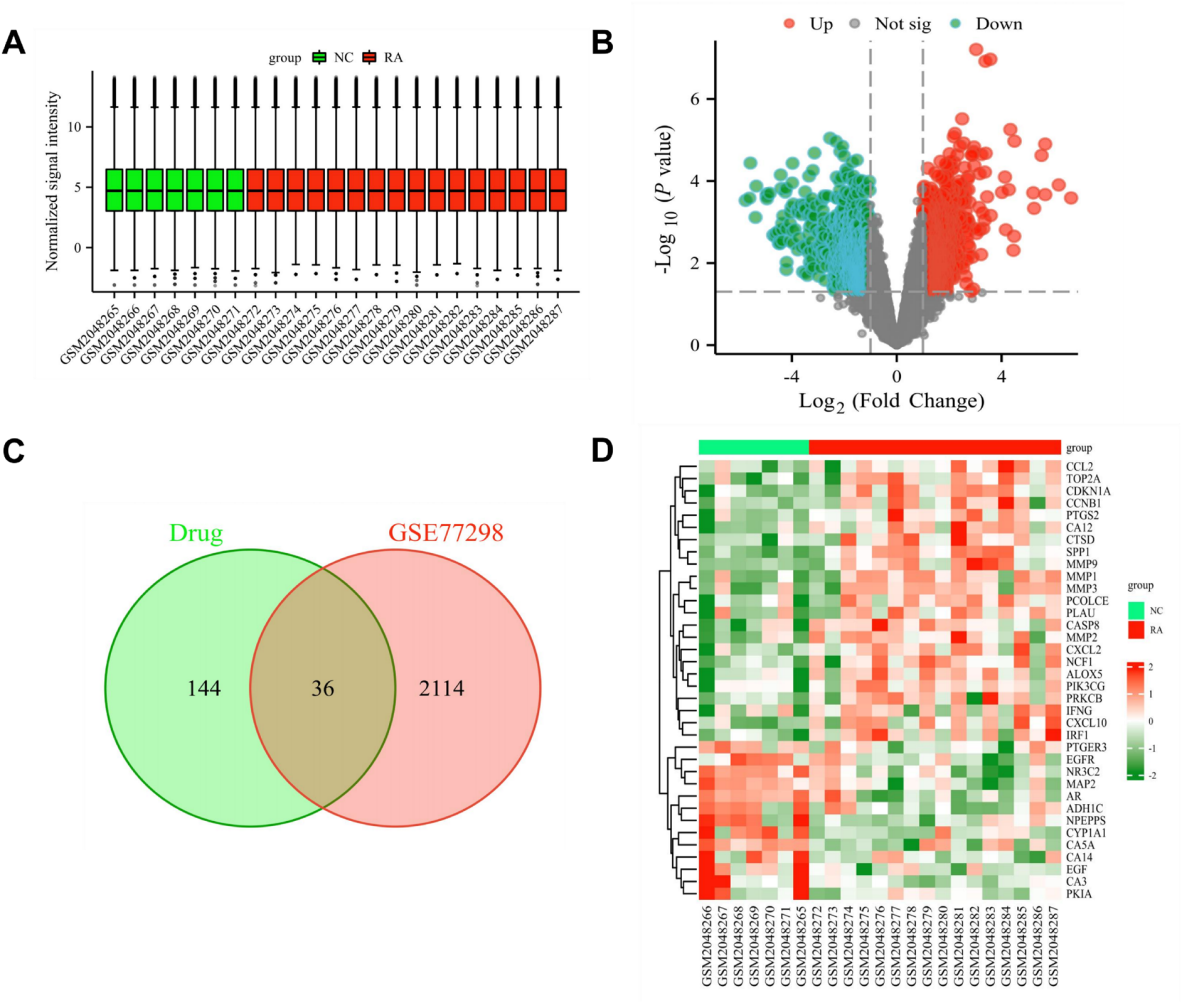
HDW-regulated differentially expressed genes identified in the datasets GSE77298. (**A**) Box diagram of GSE77298 data set, there is no outlier in the corrected sample data. (**B**) Volcano plot representing differentially expressed genes: grey indicates genes with no difference, red represents upregulated genes, and green denotes downregulated genes. (**C**) Intersection targets of disease and drug. (**D**) Heatmap showcasing differentially expressed genes. The x-axis represents samples from the two datasets, while the y-axis displays the differentially expressed genes. Red indicates high expression and green signifies low expression.

Based on the threshold |log(FC)|≥ 1 and *p* value < 0.05, we employed the "limma" package in R to identify 2150 differentially expressed genes (DEGs) in the GSE77298 dataset. Among these, 1170 genes were upregulated and 980 genes were downregulated. The box plot of the GSE77298 dataset is shown in Fig. 2A. The filtered differentially expressed genes are depicted in a volcano plot (Fig. 2B) using R. Using a Venn diagram, we identified 36 overlapping targets between HDW drug targets and RA differentially expressed genes (Fig. 2C). Heat maps showcasing the differential expression of these 36 shared targets are presented in Fig. 2D.

### Active ingredients and potential targets of HDW

We conducted the network pharmacology prediction of HDW based on the Network Pharmacology Evaluation Method Guide‐Draft. 142 HDW-related components were retrieved from TCMSP and published literature. Based on pharmacokinetic characteristics (OB ≥ 30%, DL ≥ 0.18) and ADME information, 11 major active ingredients were screened from 142 ingredients. The TCMSP and Swiss Target Prediction databases were used to determine the pharmacological targets of each component of HDW. Details of these active ingredients and targets are listed in Supplement Table S1. Finally, after removing duplicates, we identified 180 potential targets using the Uniprot database.

### Construction of disease-drug-active ingredient-target network diagram

**Figure 3:**
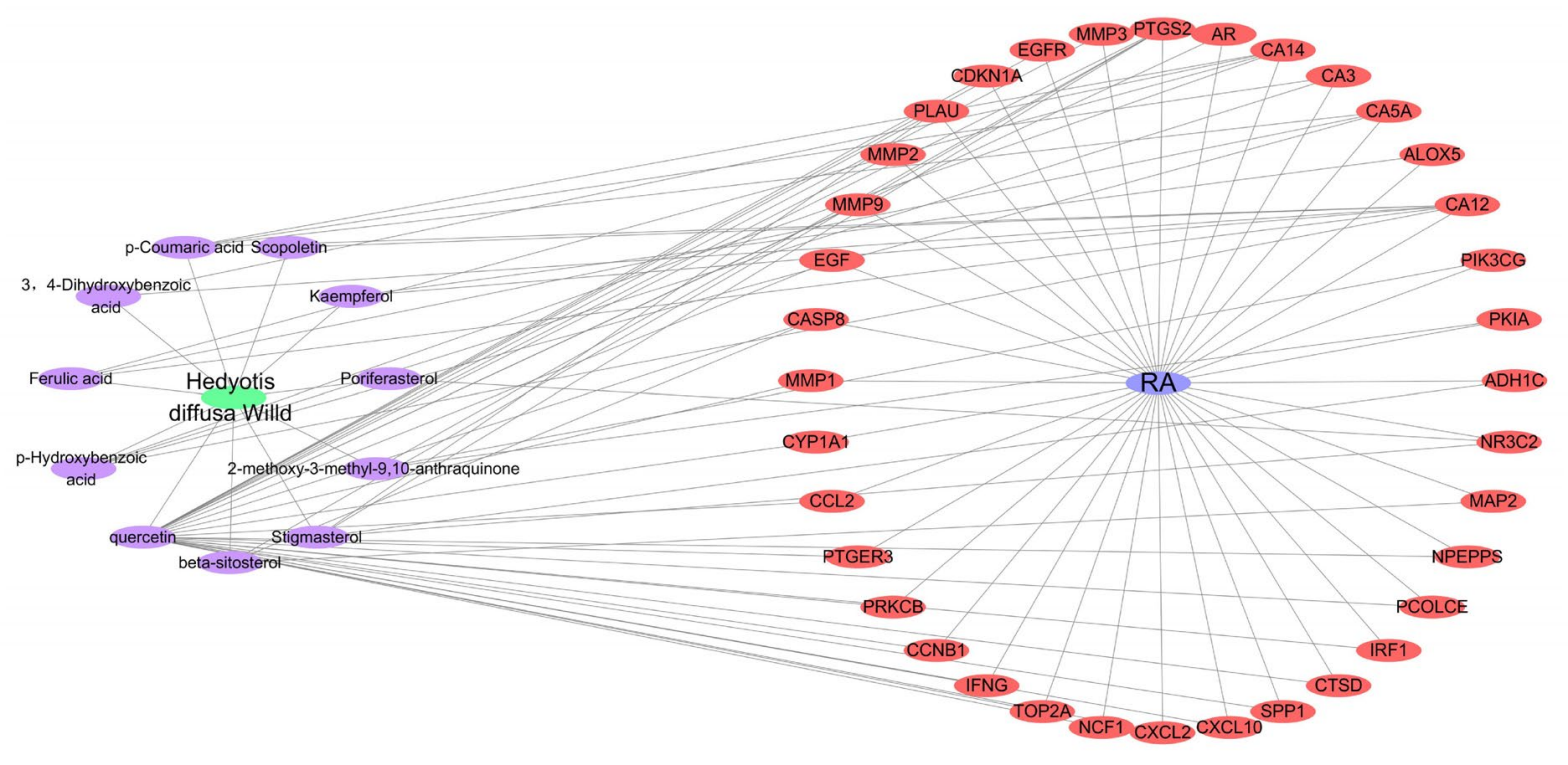
Construction of disease-drug-active ingredient-target network diagram.

We imported 11 active ingredients and 36 common targets into Cytoscape 3.9.0 software to build a RA-drug-ingredient-target network, as shown in Fig. 3.

### GO and KEGG enrichment analysis

**Figure 4:**
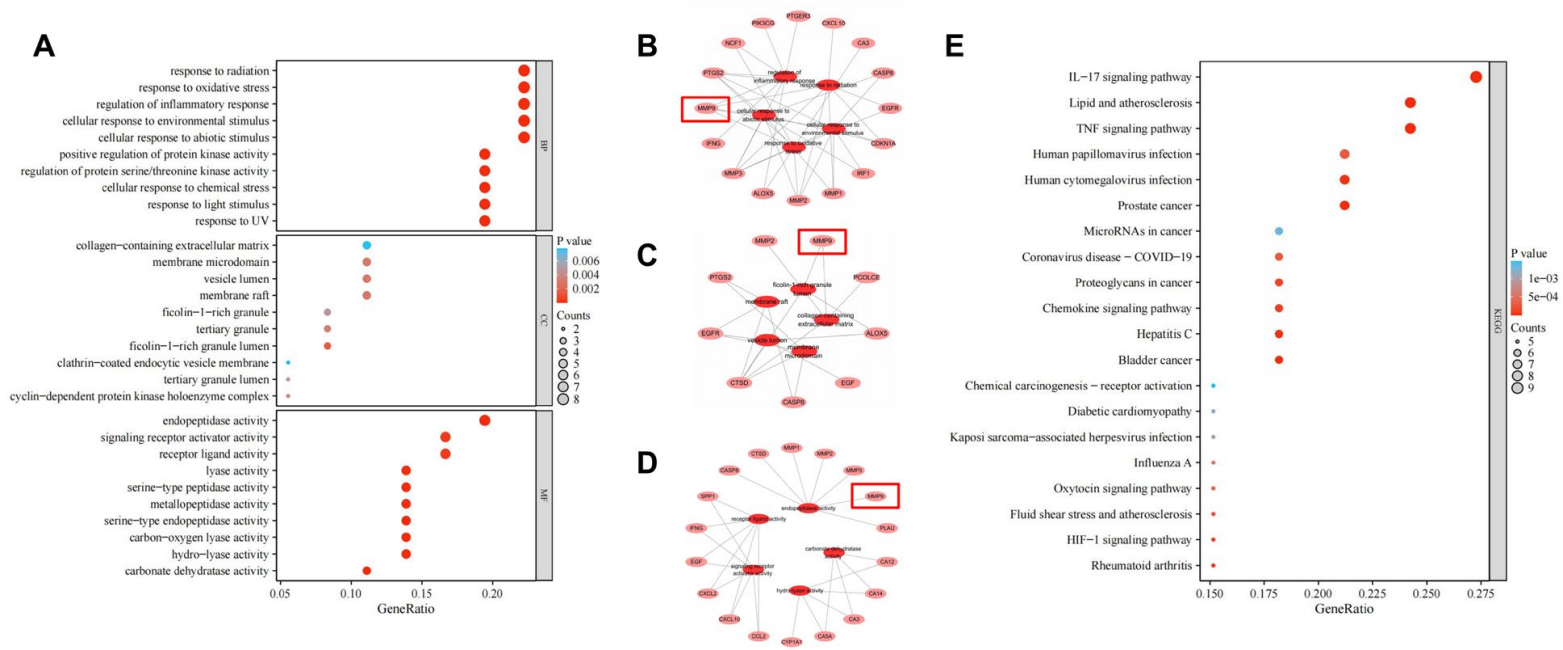
Functional analysis of differentially expressed genes. (**A**) GO enrichment analysis. The GO enrichment analysis results showed that organic substances, chemical stimuli and chemicals were significantly enriched in RA. **(B**–**D)** The top five BP, CC, MF related gene network diagrams,they all involve the gene MMP9. (**E**) KEGG enrichment analysis. KEGG results showed that IL-17, TNF signaling pathways and HIF-1 signing pathway were significantly enriched in RA.

GO analysis identified a total of 799 significantly enriched GO items (Benjamini–Hochberg corrected *p* < 0.01), which comprised 723 biological processes, 10 cellular components, and 66 molecular functions. We screened the top 10 GO terms, as shown in Fig. 4A. In the biological processes (GO:BP) category, the top terms relate to responses to radiation, oxidative stress, and regulation of inflammatory responses. In the cellular component (GO:CC) category, the most popular terms include collagen-containing extracellular matrix, membrane microdomain, and vesicle cavity. In the molecular function (GO:MF) category, the top terms include endopeptidase activity, signaling receptor activation activity, and receptor ligand activity. We further mapped the gene networks related to the top 5 BP, CC, and MF terms (Figs. 4B-4D). We observed that these top 5 GO terms were closely associated with the core gene MMP9, which is a focus of our subsequent research. To further determine the potential signaling pathway, we analyzed the KEGG pathway. Figure 4E shows the first 20 significantly enriched pathways (*p* value < 0.01). Supplementary Table S2 provides a list of genes associated with 20 selection pathways. Many targets have been found to be associated with IL17 and TNF signaling pathways, which are related to the pathogenesis and prognosis of RA.

### Screening of HDW anti-RA hub targets

**Figure 5:**
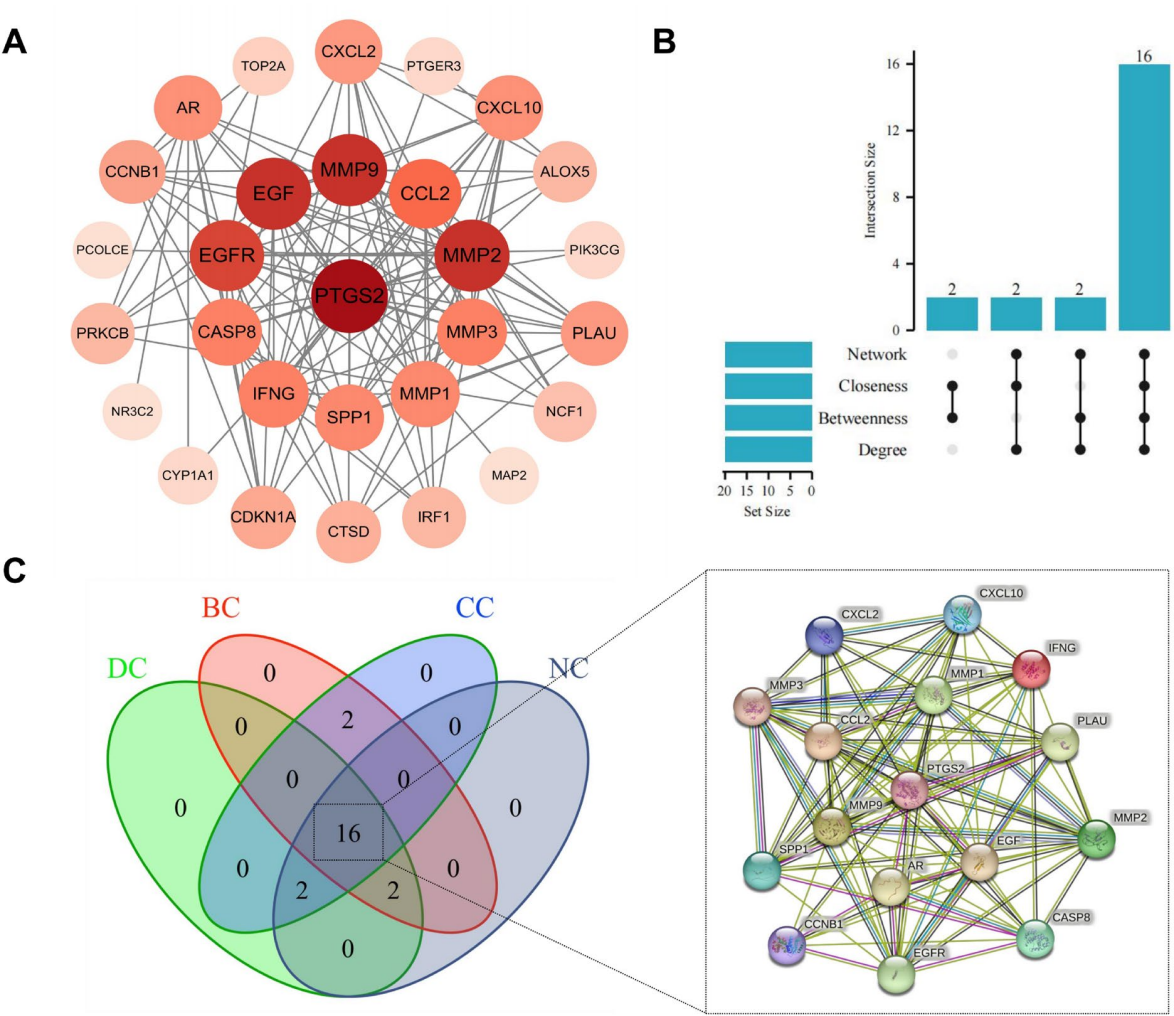
Screening hub targets through PPI network. (**A**) The PPI network of 36 common targets is drawn by cytoscape. The darker the color and the larger the shape, the higher the Degree value. (**B**) Screening the key genes in the top 20 genes by UPset map. (**C**) Key genes were screened from the PPI network using the Betweenness (BC), Closeness (CC), Degree (DC), and Network (NC) methods,sixteen key genes were screened out and their string network diagrams were drawn.

Next, we analyzed 36 potential therapeutic targets using the STRING database to obtain a PPI network to explore the relationship between RA-related targets. A PPI relationship network with 36 nodes and 139 edges was generated, with an average nodality of 7.72 (Supplementary Fig. S1). Import the PPI network diagram into the Cytoscape 3.9.0 software for visualization (Fig. 5A). We further identified subnetwork and hub targets from the PPI network using the CytoNCA plugin (Fig. 5B-5C). Supplemental Fig. S1 identifies a subnetwork consisting of 16 nodes and 92 edges. These 16 interaction targets were identified as key targets for HDW therapy for RA (Supplementary Table S3).

**Figure 6:**
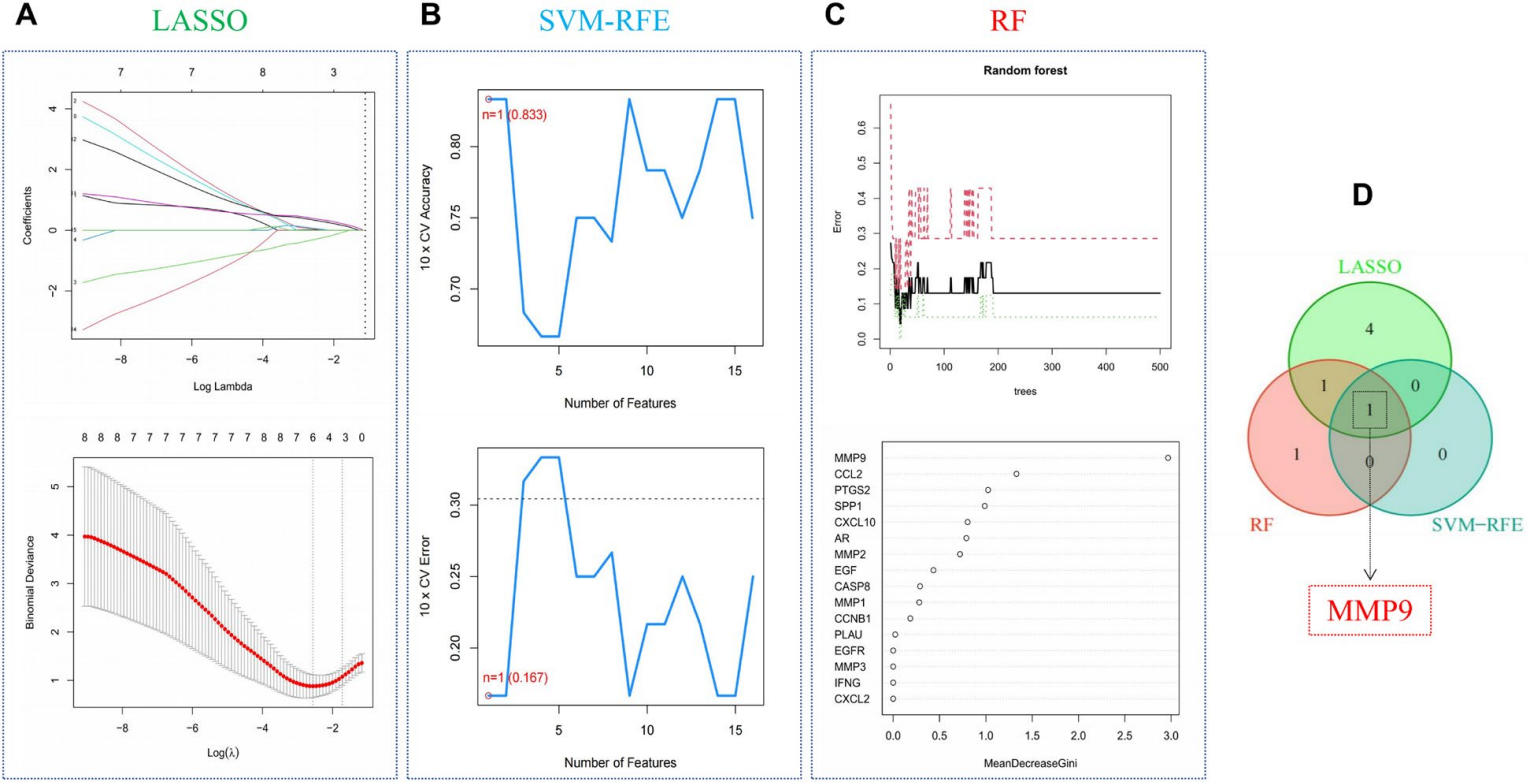
MMP9 was identified as the core gene of RA through three computational learning algorithms. (**A**) Penalized parameter adjustment by LASSO logistic regression algorithm with tenfold cross-validation was used to select 6 RA-related features. (**B**) SVM-RFE algorithm to filter 16 key genes to determine the best combination of key genes. Finally, 1 gene (maximum precision = 0.833, minimum RMSE = 0.167) was identified as the best key gene. (**C**) Key gene screening was performed by random forest algorithm, and 3 genes were identified as key genes based on gene importance greater than 1.0. (**D**) Key gene MMP9 obtained from LASSO, SVM-RFE and RF models.

One of the key genes was identified as the core gene for HDW treatment of RA. We screen meaningful key genes in the training dataset through three machine learning algorithms (LASSO, SVM-RFE, and RF) to distinguish RA samples. The LASSO algorithm selected 6 genes from 16 key genes (Fig. 6A). At the same time, in the SVM-RFE algorithm, we identified a key gene (maximum accuracy = 0.833, minimum RMSE = 0.167) (Fig. 6B). The RF algorithm was used to determine the importance of key genes, and we identified 3 genes with an importance greater than 1.0 bits (Fig. 6C). The concrete results of the three algorithms are in Supplementary Table S4. Finally, we combined the results of three machine learning algorithms to identify a core gene (MMP9, Fig. 6D).

### ROC diagnosis, GSEA enrichment analysis and immune cell infiltration analysis

**Figure 7:**
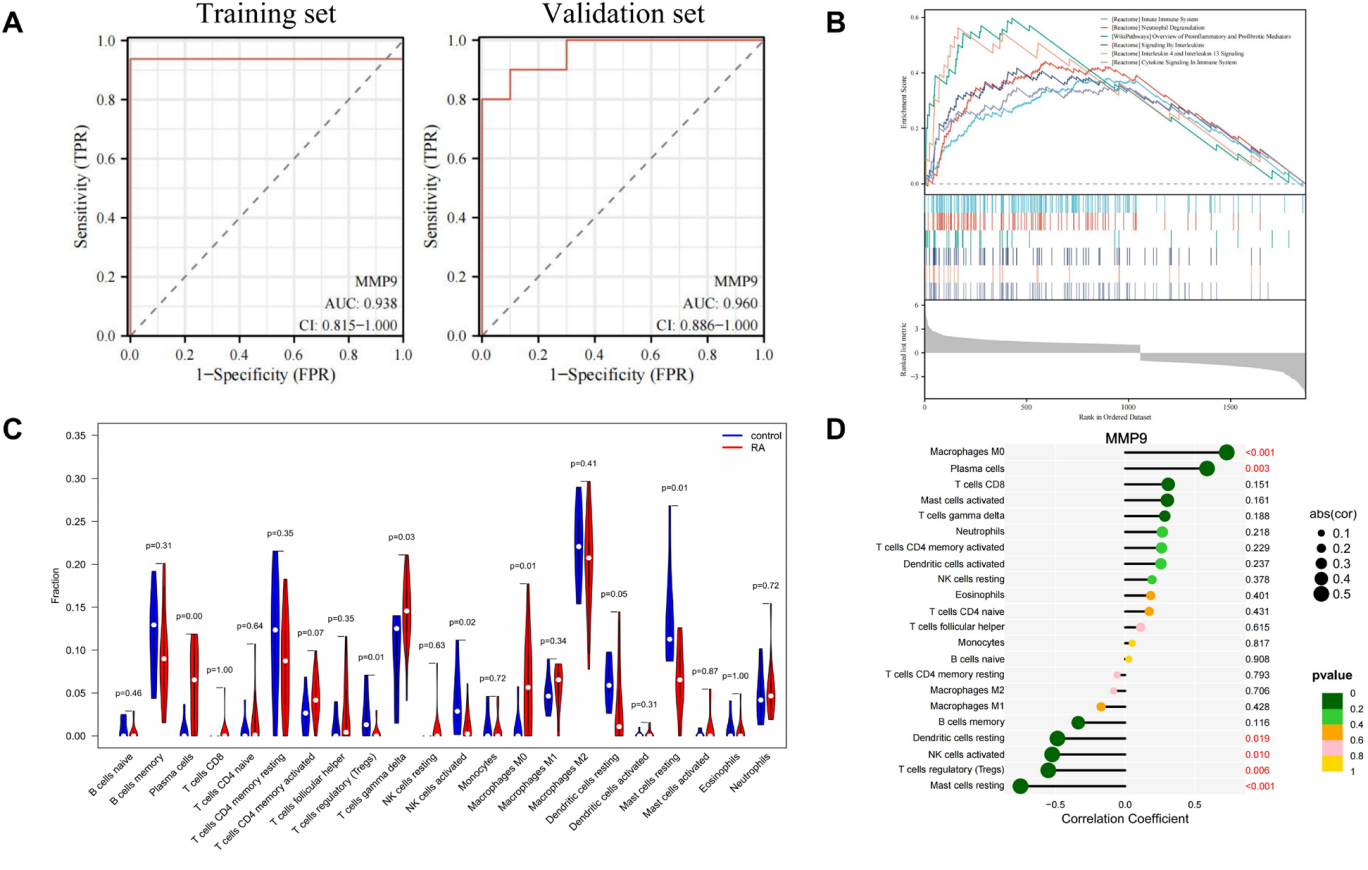
Correlation analysis of MMP9 with RA. (**A**) ROC curves of MMP9 in the training set and the validation set. (**B**) GSEA analysis of core gene MMP9. (**C**) Implementation of the CIBERSORT algorithm to explore the differences in immune microenvironment between RA and normal samples. (**D**) Graph of core gene and immune cell correlation analysis. MMP9 is almost significantly correlated with the microenvironment of differential expression obtained by immune infiltration analysis.

To investigate whether the core gene can be used to distinguish between RA samples and healthy control samples, we plotted ROC curves. As shown in Fig. 7A, the AUC of the core gene is greater than 0.9. In addition, we plotted the ROC curve of the validation dataset (GSE55235) to further validate the diagnostic potential of the core gene. The results showed that the AUC value of the core gene in the verification dataset was greater than 0.9, indicating that the core gene had certain accuracy and specificity for distinguishing RA samples from normal control samples. The above evidence suggests that the core gene MMP9 has high accuracy and specificity in distinguishing RA patients from healthy control samples.

We used GSEA analysis to understand the role and significance of core genes in RA. The first 6 pathways associated with core gene enrichment are shown in Fig. 7B. Comprehensive analysis found that MMP9 was mainly involved in immune response (innate immune system, neutrophil degranulation, and overview of pro-inflammatory and profibrotic mediators) and multiple signaling pathways (interleukin signaling, cytokine signaling).

In addition to the complex pathogenesis, RA is greatly influenced by the immune system. To investigate the differences in immune microenvironment between RA samples and healthy control samples, we employed the CIBERSORT algorithm. As shown in Fig. 7C, the expression of plasma cells, T cells gamma delta and macrophage M0 in proportional RA samples was higher than that of healthy controls, while the expression of regulatory T cells, NK cell activation and mast cell resting was lower than that of healthy controls. In addition, we analyzed the relationship between high and low expression of characteristic genes and the immune microenvironment. MMP9 was significantly positively correlated with immune macrophages M0 and plasma cells, but negatively correlated with dendritic cell rest, NK cell activation, regulatory T cells, and mast cells resting (Fig. 7D). In summary, MMP9 may strongly affect the immune microenvironment in RA patients.

### Molecular docking

**Figure 8:**
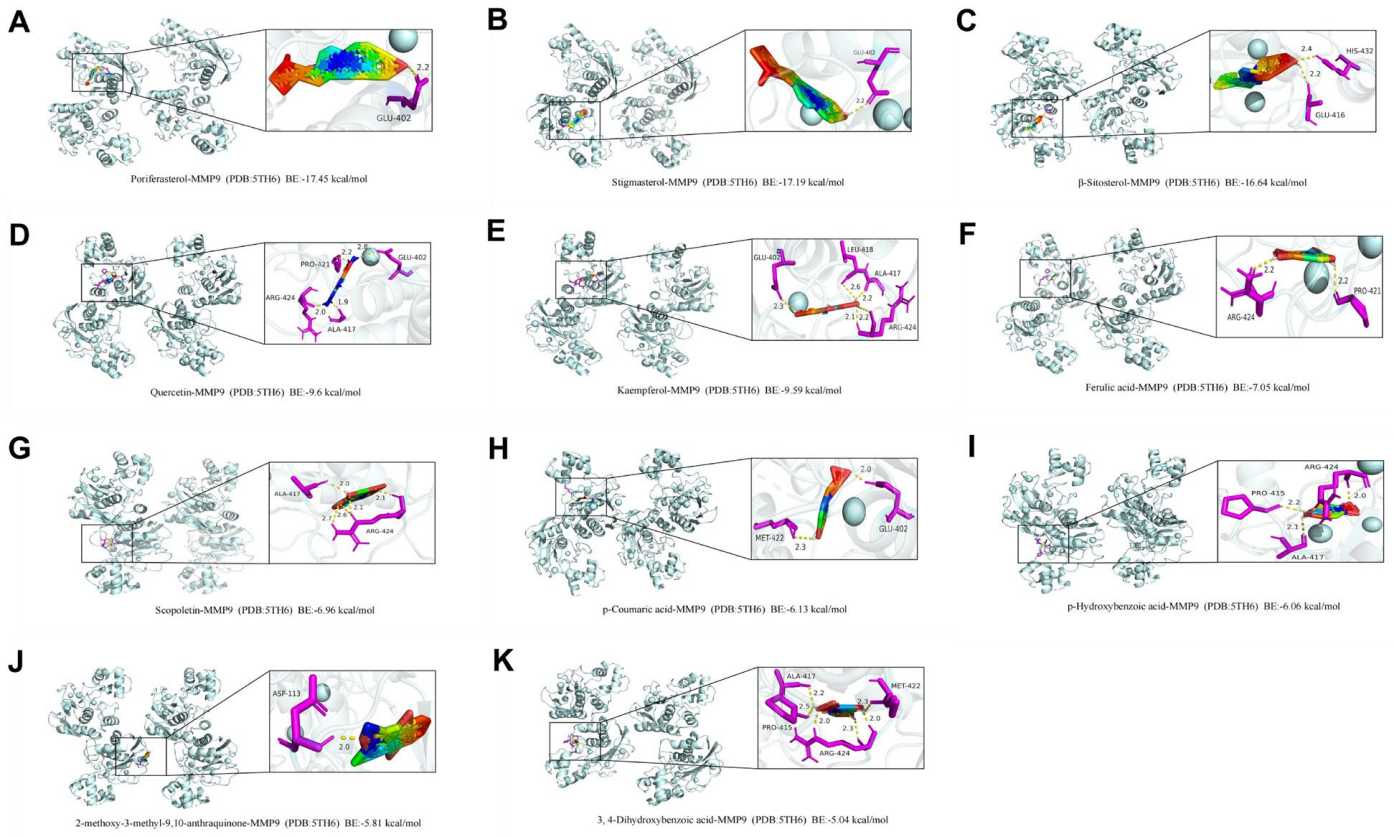
Docking complexes of ligand and receptor proteins and their binding residues are shown using PYMOL software. (**A**) MMP9 and Poriferasterol. (**B**) MMP9 and stigmasterol. (**C**) MMP9 and β-sitosterol. (**D**) MMP9 and Quercetin. (**E**) MMP9 and Kaempferol. (**F**) MMP9 and Ferulic acid. (**G**) MMP9 and Scopoletin. (**H**) MMP9 and p-Coumaric acid. (**I**) MMP9 and p-Hydroxybenzoic acid. (**J**) MMP9 and 2-methoxy-3-methyl-9,10-anthraquinone. (**K**) MMP9 and 3,4-Dihydroxybenzoic acid.

Candidate compounds 2-methoxy-3-methyl-9,10-anthraquinone, Poriferasterol, stigmasterol, β-sitosterol, Quercetin, Kaempferol, Scopoletin, p-Coumaric acid, 3,4-Dihydroxybenzoic acid, Ferulic Acid and p-hydroxybenzoic acid are the 11 main active ingredients of HDW. After molecular docking of 11 compounds with the core gene MMP9 (PDB:5TH6), the target protein and small molecules with strong binding affinity were visualized by PyMoL software (Fig. 8). The 11 components of HDW have strong binding to the core target MMP9, of which Poriferasterol has the highest binding energy. These results suggest that HDW treatment may bind to MMP9 and influence the development of RA.

### LncRNA-miRNA-mRNA axis prediction and construction

**Figure 9:**
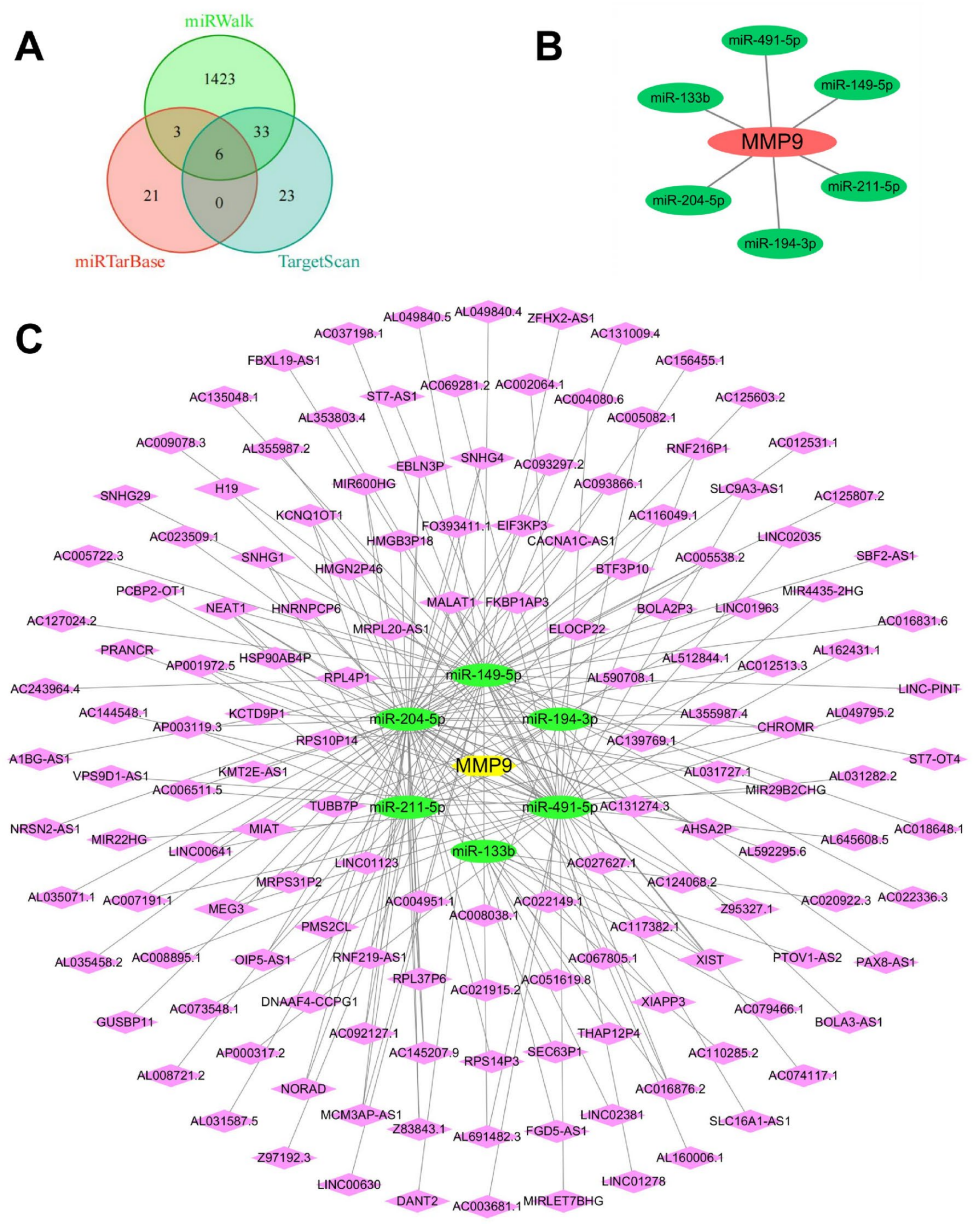
Prediction and construction of lncRNA-miRNA-mRNA axis. (**A**) MMP9 predicted a total of 6 common miRNAs in the three databases TargetScan, miRTarBase and miRWalk. (**B**) MMP9 predicted a total of 6 targeted miRNAs in the miRWalk, miRTarBase and TargetScan databases. (**C**) 147 targeted lncRNAs predicted by 6 miRNAs in the starBase and lncBase database. We used Cytoscape to construct the ceRNA network.

**Figure 10:**
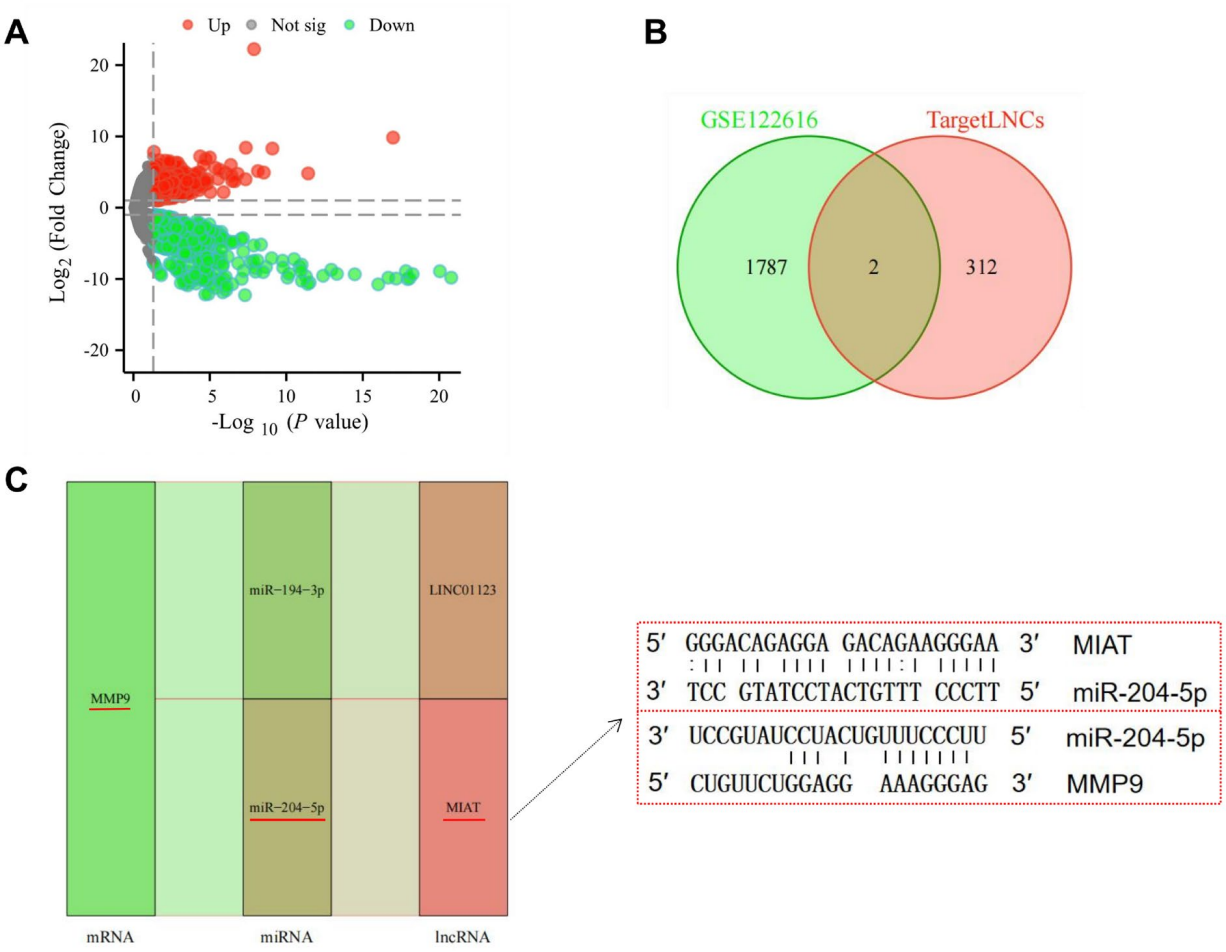
Differential lncRNAs expression analysis of GSE122616 and screened the mRNA–miRNA–lncRNA axis. (**A**) Volcanomap, black is the lncRNAs with no difference, red is the lncRNAs that are upregulated, and green is the lncRNAs that are down-regulated. (**B**) The intersection of differentially expressed genes and differentially expressed lncRNAs obtained by GSE122616 and targeted lncRNAs in ceRNA network was obtained to obtain two lncRNAs. (**C**) According to two lncRNAs and their corresponding miRNAs and mRNAs, 2 mRNA–miRNA–lncRNA axes are obtained. Predicting the binding sites of MMP9 and miR-204-5p, miR-204-5p and MIAT.

MMP9 predicts target miRNAs in the TargetScan, miRTarBase, and miRWalk databases, and the intersection of the three databases yields a total of 6 miRNAs (Fig. 9A-B). LncRNAs targeting 6 miRNAs were predicted using starBase and lncBase databases, and after removing duplicate lncRNAs, 6 miRNAs predicted 147 target lncRNAs. Cytoscape was used to construct a ceRNA network based on 147 lncRNAs, 6 miRNAs and MMP9 (Fig. 9C). GSE122616 obtained a volcano map (Fig. 10A) by R visualization after differential analysis and 1789 DElncRNAs were obtained. DElncRNAs obtained from GSE122616 cross with MMP9-targeted lncRNAs to generate two lncRNAs (Fig. 10B). Based on the ceRNA network, we obtained MMP9-miR-204-5p-LINC01123 and MMP9-miR-204-5p-MIAT two axes associated with the pathogenesis of RA (Fig. 10C). Through preliminary experiments, it was found that HDW treatment had no obvious regulatory effect on LINC01123 but could significantly affect the expression of MIAT, so we chose MIAT for subsequent experiments.

### Cell experimental verification

**Figure 11:**
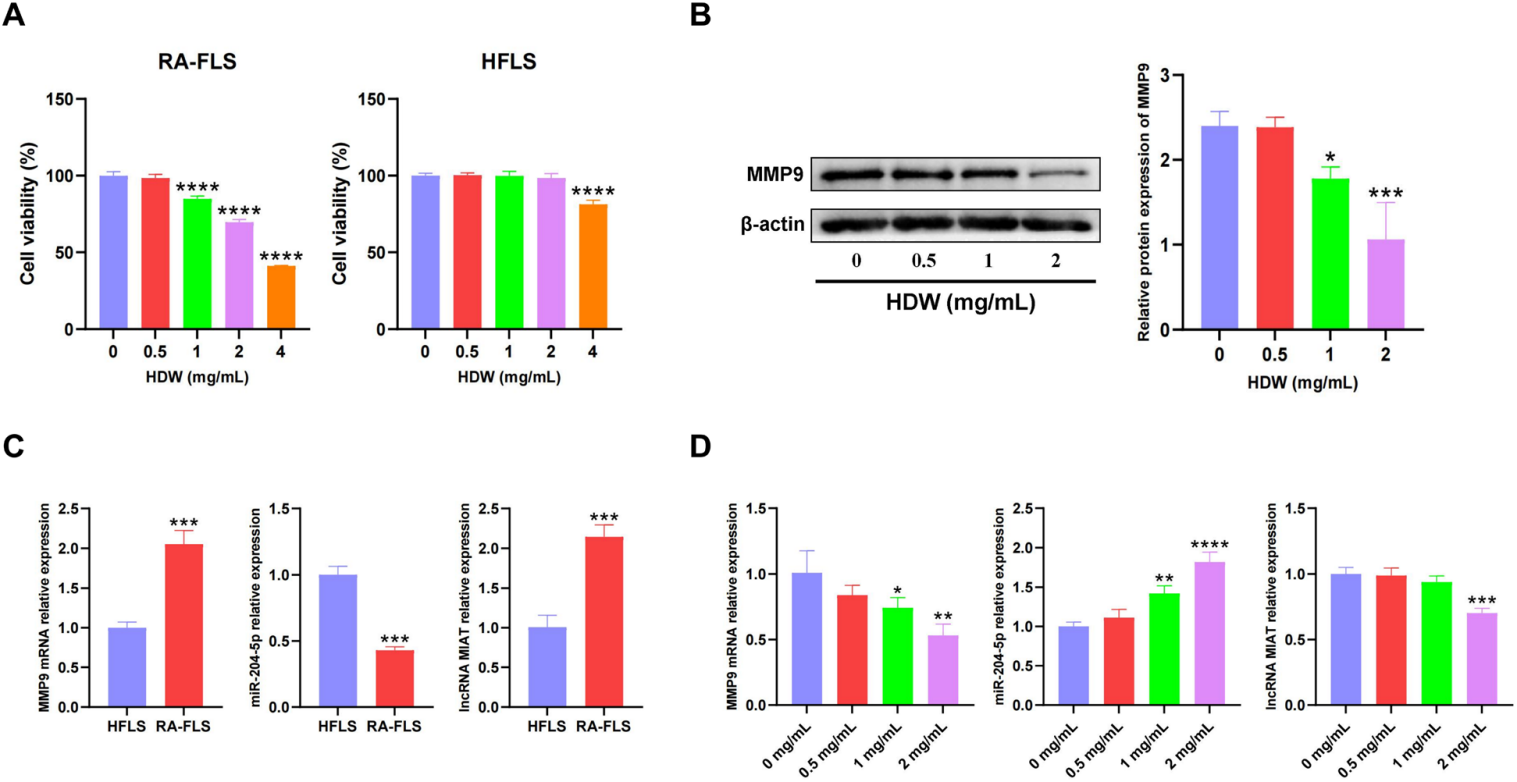
Cell experiments validate results of network pharmacology and bioinformatic analysis. (**A**) CCK8 assays of different HDW concentrations (0, 0.5, 1, 2, 4 mg/mL) incubated RA-FLS and HFLS cells for 48 h. (**B**) The expression levels of MMP9 was measured using western blotting. (**C**) The expression levels of MMP9, miR-204-5p and MIAT was measured using RT-PCR in HFLS and RA-FLS. (**D**) The effect of HDW on the mRNA levels of MMP9, miR-204-5p and MIAT in RA-FLS. compared with control (0 mg/mL), *p < 0.05, **p < 0.01, ***p < 0.001, ****p < 0.0001.

Over-proliferation of RA-FLS is an important pathogenesis of RA. In this study, the effects of different concentrations (0, 0.5, 1, 2, 4 mg/mL) of HDW on the proliferation of RA-FLS cells and HFLS cytotoxicity after 48 h were studied. CCK-8 experiments showed that HDW inhibited the proliferation of RA-FLS cells in a dose-dependent manner and cytotoxicity to HFLS at 4 mg/mL (Fig. 11A). The results of IC50 assay showed that the 50% inhibition rate of the drug was 3.225 mg/mL (Supplement Fig. S2), considering that 4 mg/mL already had high cytotoxicity, so we selected doses of 0.5, 1, and 2 mg/mL for follow-up experiments. WB results showed that HDW could reduce MMP9 expression in RA-FLS cells in a dose-dependent manner (Supplement Fig. S3 and Fig. 11B). Some edges of the original strip may not be reflected due to the developer, but after three repeated experiments, we can guarantee the authenticity and repeatability of the data. We verified the expression of MMP9/miR-204-5p/MIAT in RA-FLS by qRT-PCR, and the results showed that MMP9 and MIAT were highly expressed, and miR-204-5p expression was down-regulated (Fig. 11C), which was consistent with the results of previous literature. Finally, we verified the changes of MMP9/miR-204-5p/MIAT expression after HDW treatment by qRT-PCR, and the results showed that MMP9 and MIAT expression were down-regulated and miR-204-5p expression was upregulated (Fig. 11D). These results validate our network pharmacological analysis and suggest that HDW may influence the occurrence and development of RA by regulating the MMP9/miR-204-5p/MIAT axis.

## Discussion

HDW exhibits anti-inflammatory, antitumor, and immunomodulatory effects. It demonstrates significant anti-inflammatory therapeutic outcomes in inflammatory model therapies for systemic lupus erythematosus and osteoarthritis^24^. However, the role of HDW in RA is seldom reported, and its specific ceRNA regulatory axis in treating RA remains unexplored. In this study, using network pharmacology, bioinformatics, and molecular docking methods, we identified MMP9 as the core gene targeted by HDW for RA. Then, according to the mechanism of action of ceRNA, we screened and predicted the ceRNA axis MMP9/miR-204-5p/MIAT targeting RA diseases of HDW. Cell experiments confirmed that HDW inhibits RA-FLS proliferation and MMP9 protein expression in a concentration-dependent manner, further regulating gene expression within the MMP9/miR-204-5p/MIAT network axis. Therefore, through a novel integrated biological screening approach, we identified the ceRNA regulatory network axis targeted by HDW for RA.

In this study, the active ingredient of HDW and target MMP9 in the treatment for RA were clarified for the first time through network pharmacology combined with bioinformatics analysis. Historically, disease-related genes were collected mainly through multiple online databases (OMIM, DrugBank, TTD, GeneCards, and Dis-GeNET databases) without tissue specificity. We detected RA differential gene expression using the GEO database GSE77298, focused on the synovial fluid chip. The number of genes identified exceeded those found on online databases, exhibited a stronger correlation, and proved more relevant for inflammation-related phenotypes and signaling pathways when viewed from an enrichment perspective. Compared with previous studies of RELA, TNF, IL-6, the intersection genes were mainly in MMP1, MMP2, MMP3, MMP9, EGF and EGFR^18^. While genes related to inflammation from online databases appear scattered, the data analyzed from RA patients using a chip demonstrates distinct individual patterns, revealing a gene network aligned with the patient's pathological phenotype. The pathology of GSE77298 RA patients may be related to immune cell invasion, cartilage destruction and the formation of angiogenesis. It is worth noting that there are many relevant GEO datasets about RA, and the choice of GEO dataset will affect the analysis results. While considering the representativeness and temporality of the dataset, it is necessary to pay attention to the source and detection method of the dataset samples. In the analysis of the same type of GEO dataset, we used GSE77298 as the main test analysis set, and also used the GSE55235 as the verification level for analysis, and obtained the same results, and determined that the key gene for HDW action was MMP9 (Supplement Figs. S4, S5, and S6). At the same time, where possible, multiple GEO datasets can be used to intersect to enhance the broad-spectrum applicability of data analysis.

Pro-inflammatory cytokines and matrix metalloproteinases (MMPs) play an important role in the proliferation, migration and invasion of RA-FLS and even the erosion of articular cartilage^25^. MMP9 is a glycosylated collagenase in matrix metalloproteinases, whose main function is to maintain the homeostasis of the extracellular matris. It degrades types I, IV, V, and X collagen and fibrobinin, laminin and proteoglycans in the extracellular matrix, which are important components of articular cartilage. The main pathological feature of RA is the infiltration of a variety of inflammatory cells, and the immune cells in the synovial cavity produce pro-inflammatory factors and MMPs, which act on chondrocytes, which in turn cause bone tissue destruction, and eventually lead to joint deformity and loss of function. It can be seen that MMP9 is an important point in the pathogenesis of RA. MMP9 has been shown to have multiple roles in key pathological processes such as angiogenesis, inflammation, and cytokine activation^26,27^. MMP9 can promote the aggregation and activation of inflammatory cells, thus promoting the occurrence and development of inflammatory reaction. MMP9 can degrade articular cartilage and bone tissue, thus promoting the occurrence of joint destruction and deformity^28,29^. MMP9 is involved in the regulation of mitochondrial related functions and the progression of COVID-19 disease, and may become a potential therapeutic target for severe COVID-19^30^. Synovial fluid of rheumatoid arthritis contains high levels of MMP-9, including its truncated and citrullinated protein forms. The combination of MMP-9 as an analyte and PTM through citrullinated may have clinical significance, especially in the field of arthritis diseases^31^. MMP9 can be expressed in RA-FLS, MMP9 contributes to the proliferation, migration and invasion of RA-FLS^32^, and reducing its production can effectively inhibit RA-FLS-mediated cartilage degradation, so MMP9 expression detection can effectively predict the progression of bone destruction^33^. Our ROC analysis found that MMP9 has good diagnostic value in RA, GSEA enriched it to participate in various immune responses and multiple inflammatory signaling pathways. Immune infiltration analysis found that MMP9 may strongly affect the immune microenvironment of RA patients. Molecular docking also confirmed that MMP9 and 11 active ingredients of HDW have good binding activity. In this study, RT-PCR and WB experiments showed that MMP9 was highly expressed in RA, and HDW could reduce the expression of MMP9 in RA-FLS in a concentration-dependent manner. These experiments proved the above network pharmacological predictions, and showed that HDW may affect the occurrence and development of RA by regulating the expression of MMP9. As a member of the inflammatory network gene, MMP9 is affected by the regulation of many upstream genes, so the regulatory role of ceRNA based on MMP9 deserves further study.

As a new regulatory mechanism, ceRNA related researches have shown that drugs can affect the occurrence and development of diseases by regulating the ceRNA axis. Yang et al.^34^ found that baicalin could affect the ceRNA regulatory network lncRNA ENSR-NOT00000076420/miR-144-3p/Fosb, lncRNA MSTRG.1448.13/miR-144-3p/Atp2b2 and lncRNA MSTRG.1448.13/miR-144-3p/Shanks alleviated joint lesions in CIA rats. Wen et al.^35^ showed that Triptolide inhibits the growth and inflammatory response of RA-FLS by regulating the hsa-circ-0003353/microRNA-31-5p/CDK1 axis. Based on the ceRNA hypothesis of MMP9, two lncRNAmiRNA-mRNA axes for the treatment of RA were preliminarily constructed, namely MMP9/miR-194-3p/LINC01123 and MMP9/miR-204-5p/MIAT. After we treated RA-FLS cells with a certain concentration gradient of HDW, we found that the expression of LINC01123 did not change, but the expression of MIAT changed significantly by RT-PCR. Therefore, the MMP9/miR-204-5p/MIAT axis was selected as the follow-up research object in this experiment. Both MIAT and miR-204-5p have been reported to be closely associated with RA, and studies have shown that miR-204 affects synovial hyperplasia and inflammation of RA by regulating structure-specific recognition protein 1 (Ssrp1), suggesting that miR-204 can be used as a novel biomarker for the treatment of RA^36^. Xiao et al.^37^ proposed that miR-204-5p is reduced in RA synovial tissue, and miR-204-5p overexpression can effectively inhibit cell proliferation and inflammatory response, and trigger RA-FLS apoptosis. MIAT regulates the proliferation and apoptosis of chondrocytes in osteoarthritis, and silencing MIAT can inhibit cell viability, inhibit DNA synthesis, and promote apoptosis, suggesting that MIAT is involved in the development of inflammatory diseases^38^. Wang et al.^39^ confirmed that MIAT expression is higher in synovial tissue of collagen-induced arthritis (CIA) mice than that of normal synovial tissue. Their study also suggests that MIAT may be an inflammatory inhibitor, because IL-1β, TNF-α expression increases when it is knocked out in vitro. Deng et al. verified that MIAT can directly target miR-204-5p competitively bound to HMGB1 in cerebral microvascular endothelial cells after cerebral ischemia^40^. Wang et al. verified that miR-204-5p directly targeted MMP9 competitive binding through diluciferase reporter enzyme experiment in atherosclerosis-associated abnormal human vascular smooth muscle cells (hVSMC) and MCF-7 breast cancer cells^41,42^. Based on the above studies, we hypothesize that MIAT may participate as a ceRNA sponge in the regulation of miR-204-5p on the downstream gene MMP9, which in turn has an impact on the development and development of RA. The results of cell experiments showed that miR-204-5p was poorly expressed in RA-FLS and MIAT was highly expressed in RA-FLS, which was consistent with the prediction of ceRNA regulation mechanism, and the expression of miR-204-5p was concentration-dependent up-regulated and MIAT expression was concentration-dependent down-regulated after HDW treatment of RA-FLS. It suggests that HDW may directly bind or indirectly regulate MMP9, thereby affecting the MMP9/miR-204-5p/MIAT axis to participate in the occurrence and development of RA.

## Methods

### Potential targets of RA

The gene expression data (GSE77298) came from the National Central Gene Expression Comprehensive Dataset (https://www.ncbi.nlm.nih.gov/geo). The synovial tissue samples from 16 RA patients and 7 healthy donors were selected in GSE77298. We used R language (version 4.2.1) and related R packages (ComplexHeatmap[2.13.1]) to analyze the differentially expressed genes in the selected samples in the bioinformatics tool software of Xiantao Academic (https://www.xiantaozi.com/), and the results were screened by *p* value < 0.05 and |logFC|≥ 1 to obtain potential targets for RA.

### Active ingredients and potential targets of HDW

The active ingredients and potential targets of HDW were selected from the Traditional Chinese Medicine Systems Pharmacology Database and Analysis Platform (TCMSP, https://old.tcmsp-e.com/tcmsp.php)^43^, and the screening thresholds were the recommended OB ≥ 30% and DL ≥  0.18^44^. Using both the search sites PubMed (https://pubmed.ncbi.nlm.nih.gov/) and CNKI (https://www.cnki.net/), the database SwissADME (http://www.swissadme.ch/) extended the screening of active ingredients in HDW. On this basis, the target corresponding to the active ingredient (probability ≥ 0.9) was further searched from the SwissTargetPrediction database (http://swisstargetprediction.ch/)^45^. Finally, duplicate targets are deleted to obtain potential targets for further analysis.

### Construction of disease-drug-active ingredient-target network diagrams

We used Venny 2.1.0 to intersect drug targets with potential targets of RA to obtain potential targets for the HDW treatment of RA. Then, GraphPad Prim 9.0 software was used to get the expression heatmap of potential therapeutic targets, and Cytoscape 3.9.0 software^46^ was used to establish a "disease-drug-active ingredient-target" network diagram to determine their interaction.

### Gene ontology and pathway enrichment analysis

We introduced intersection genes into the bioinformatics tools in Xiantao Academic (https://www.xiantaozi.com/) for analysis, Gene ontology (GO) and KEGG pathway analyses were conducted using R language (version 4.2.1) and related R packages (clusterProfiler, Org.Hs.eg.db, and ggplot2)^47,48^. After adjusting the *p* value using the Benjamini–Hochberg (BH) method, the *p* < 0.01 was considered statistically significant.

### Network visualization and identification of hub targets

We built a protein Interaction (PPI) network diagram in the STRING database^49^ by using the screened intersection genes. Minimum required interaction score was set to the default of 0.400, and exported a file in TSV format. Genes were sorted according to the Degree value in the Cytoscape plug-in. The bigger the circle and the darker the color, the higher the Degree value. Finally, it was visualized with Cytoscape 3.9.0. Key targets were screened using the median of Degree centrality (DC), Betweenness centrality (BC), Closeness centrality (CC) and Network centrality (NC) as cut-off points. Taking the intersection of the top 20 rankings of DC, BC, CC and NC as our key genes. The Least Absolute Shrinkage and Selection Operator (LASSO) and support vector machine (SVM) are used to classify key targets. To distinguish between RA and control samples, a tenfold cross-validation was performed using the “glmnet” 17 packet. The SVM-REF algorithm is able to generate a hyperplane with the largest boundary in the feature space to distinguish between positive and negative instances. SVM-RFE algorithm analysis was performed using the “e1071” and “svmRadial” packages in R software to screen for high-quality genes. Recursive partitioning is used to construct binary trees in random forests (RFs). An RF classification model was constructed using the "RandomForest" software package to rank key genes according to the Gini index to screen for targets for characteristic expression. The crossover genes of the three machine learning algorithms are considered to be the core genes for HDW treatment of RA.

### Core target ROC diagnosis and GSEA enrichment analysis

The “pROC” package was used to evaluate the predictive power of core target diagnosis. Calculate the AUC of the ROC to determine the accuracy of the predictive model; The higher the AUC value, the higher the accuracy of the predictive model. GSEA analysis was performed on key genes to explore their biological significance, The gene sequencing method was set by selecting the Signal2Niose value, and function using the “c2.cp.all.v2022.1.Hs.symbols” geneset from the molecular signature data channel (MSigDB, http://softw-are.broad institute.org/gsea/MSigDB).

### Immune cell infiltration analysis

CIBERSORT is an analytical tool that deconvolves the expression matrix of human immune cell isotypes based on the principle of linear support vector regression. We used the CIBERSORT algorithm (http://ciber-sortx.stanf-ord.edu) to determine the relative proportion of the 22 invading immune cells. In addition, the "Spearman" rank correlation analysis in R software is used to determine the association between infiltrating immune cells and between characteristic genes and the number of infiltrating immune cells. The graphical method of the “ggplot2” package is used to show the resulting correlation.

### Molecular docking

To investigate the association between the active ingredient and the pivotal target, we applied molecular docking analysis. Download mol2 structure files for 11 main active ingredients from TCMSP. The crystal structure of the core target is obtained from the protein database (PDB, https://www.rcsb.org/). Molecular docking studies were conducted using AutoDock 4.0 software. The grid box parameters (x center: 12.573, y center: 23.87, z center: 19.304, size x: 250, size y: 194, size z: 272) that we set when we were running autodock. The lower the affinity fraction, the better the binding effect, affinity < − 4.25 kcal·mol^−1^ indicates the presence of binding activity between ligand and target; affinity < − 5.0 kcal mol^−1^ implies good binding activity; affinity < − 7.0 kcal mol^−1^ suggests strong docking activity^50^. At last, we used the Pymol program to visualize the binding pattern.

### mRNA–miRNA–lncRNA axis prediction and ceRNA network construction

We used online databases including TargetScan (https://www.targetscan.org), miRTarBase (https://mirtarbase.cuhk.edu.cn) and miRWalk (http://mirwalk.umm.uni-heidelberg.de/) databases to predict the miRNA targeting mRNA, and intersected the miRNAs predicted by the three databases to get our core miRNA. Then we used the starBase (https://rnasysu.com/encori/) and lncBase (https://diana.e-ce.uth.gr/lncbasev3) databases to predict the lncRNAs targeting miRNA, and the results of the two databases were used to retrieve the core lncRNAs. We used Cytoscape software to draw the network diagram of the selected mRNA, miRNA, lncRNA. Then we analyzed the difference of the common dataset GSE122616, and used R to visualize the volcano diagram to obtain the lncRNAs that could be expressed differently. We intersected the DElncRNAs of the common dataset with the lncRNAs predicted by the database, and finally got the lncRNAs we wanted. The screened ceRNA networks were drawn by using Sanji diagram.

### Cell culture and treatment

RA-FLS cell line was purchased from Jennio biological technology (Guangzhou, China). It was maintained in DMEM with 10% fetal bovine serum (Gibco, USA), supplemented with 1% penicillin–streptomycin (Hyclone, USA). HDW (Lot. Number: R02S11Y123184) was purchased from Shanghai Yuanye Bio-Technology.

### Cell counting kit-8 (CCK-8) assay

CCK-8 (Biyuntian, Shanghai, China) assay was used to measure cell proliferation. RA-FLS cells were seeded into 96-well plates at a cell density of 5 × 10^3^ cells/well and incubated in a 5% CO_2_ incubator at 37 ℃. After adhering to the wall, cells were treated with HDW of different concentrations (0, 0.5, 1, 2 and 4 mg/mL) for 48 h. Optical density at 450 nm was measured with a microplate reader after adding the CCK-8 solution and incubated for additional 2 h.

### Quantitative real-time polymerase chain reaction (qPCR)

RA-FLS cells were seeded in 6-well plates at 5 × 10^5^ pcs/well, and after 48 h of HDW treatment, cells were collected by trypsinization. RA-FLS cell total RNA was extracted with Trizol reagent, cDNA was synthesized with reverse transcription kit (Bio-Rad, USA), and qPCR was performed using SYBR Green PCR Master Mix (Bio-Rad, USA). Finally, the relative expression of the target was calculated by the 2^−∆∆Ct^ method. The primers used (General Biosynthesis, China) are listed in Supplementary Table S5.

### Western blotting (WB)

RA-FLS cells are cultured in T25 cell culture flasks. After HDW treatment for 48 h, lyse cells with 1 × RIPA lysis buffer containing 1% PMSF and 1% phosphotransferase inhibitor. Protein concentration is detected using the BCA Protein Quantitative Kit (Thermo Fisher Scientific, USA). Subsequently, the protein (20 μg) is denatured by heating, electrophoresis with 10% SDS-PAGE, and transferred to a polyvinylidene fluoride membrane (Millipore, USA). This is followed by sealing with 5% skim milk at 37 ℃ for 2 h and incubating overnight at 4℃ with MMP9 primary antibody (Biyuntian, Shanghai, China, AF5234). After incubation, the membrane is incubated with horseradish peroxidase (HRP)-bound secondary antibodies (Affinity Biosciences, S0001) at 37 ℃ for 1.5 h. Finally, specific bands were detected using enhanced chemiluminescence reagent (Thermo Fisher Scientific, USA) and specific bands were calculated using Image J software for protein quantification.

### Statistical analysis

Data are presented as the mean ± standard deviation (SD). All statistical analyses were conducted utilizing GraphPad Prism 9.0. The data were analyzed using one-way ANOVA and two-way ANOVA, where *p* < 0.05 was considered statistically significant. Every experiment was repeated three times.

## Conclusions

In summary, through network pharmacology and bioinformatics analysis, we constructed the MMP9/miR-204-5p/MIAT transcription network of HDW for RA. Cell experiments verified the expression levels of MMP9, miR-204-5p, and MIAT after HDW treatment of RA-FLS. It was established that HDW inhibits MMP9 protein expression and may regulate the MMP9/miR-204-5p/MIAT axis, influencing the onset and progression of RA. However, further investigation is needed to determine whether HDW regulates other RA phenotypes through this ceRNA action axis and to assess its therapeutic effect on animal models of RA.

### Supplementary Information

Supplementary Information.

## Data Availability

All data generated or analysed during this study are included in its supplementary information files.

## References

[1] Jang S, Kwon E-J, Lee JJ (2022). Rheumatoid arthritis: Pathogenic roles of diverse immune cells. Int. J. Mol. Sci..

[2] Chemin K, Gerstner C, Malmström V (2019). Effector functions of CD4^+^ T cells at the site of local autoimmune inflammation-lessons from rheumatoid arthritis. Front. Immunol..

[3] Degboé Y (2019). Polarization of rheumatoid macrophages by TNF targeting through an IL-10/STAT3 mechanism. Front. Immunol..

[4] Wehr P, Purvis H, Law S-C, Thomas R (2019). Dendritic cells, T cells and their interaction in rheumatoid arthritis. Clin. Exp. Immunol..

[5] Bergot A-S, Giri R, Thomas R (2019). The microbiome and rheumatoid arthritis. Best Pract. Res. Clin. Rheumatol..

[6] Grillet B (2023). Matrix metalloproteinases in arthritis: Towards precision medicine. Nat. Rev. Rheumatol..

[7] Abbasi M (2019). Strategies toward rheumatoid arthritis therapy; The old and the new. J. Cell. Physiol..

[8] Shirai T (2023). Celastrol suppresses humoral immune responses and autoimmunity by targeting the COMMD3/8 complex. Sci. Immunol..

[9] Li W (2021). Disease-modifying anti-rheumatic drug prescription baihu-guizhi decoction attenuates rheumatoid arthritis via suppressing toll-like receptor 4-mediated NLRP3 inflammasome activation. Front. Pharmacol..

[10] Chen R, He J, Tong X, Tang L, Liu M (2016). The *Hedyotis*
*diffusa* Willd. (Rubiaceae): A review on phytochemistry, pharmacology quality control and pharmacokinetics. Molecules.

[11] Li Y (2022). The protective capability of *Hedyotis*
*diffusa* Willd on lupus nephritis by attenuating the IL-17 expression in MRL/lpr mice. Front. Immunol..

[12] Wazir J (2021). The effectiveness of *Hedyotis*
*diffusa* Willd extract in a mouse model of experimental autoimmune prostatitis. Andrologia.

[13] Li H (2019). *Hedyotis*
*diffusa* Willd. inhibits VEGF-C-mediated lymphangiogenesis in colorectal cancer via multiple signaling pathways. Oncol. Rep..

[14] Wu K (2022). Inhibitory effects of total triterpenoids isolated from the *Hedyotis*
*diffusa* Willd on H1975 cells. Front. Pharmacol..

[15] Xu X (2021). Exploring the mechanisms of anti-ovarian cancer of *Hedyotis*
*diffusa* Willd and *Scutellaria*
*barbata* D Don. through focal adhesion pathway. J. Ethnopharmacol..

[16] Wang JH, Shu LH, Yang LL, Zhang M, He P (2011). 2-Hydroxy-3-methylanthraquinone from *Hedyotis*
*diffusa* WILLD induces apoptosis via alteration of Fas/FasL and activation of caspase-8 in human leukemic THP-1 cells. Arch. Med. Res..

[17] He J (2018). Scandoside exerts anti-inflammatory effect via suppressing NF-κB and MAPK signaling pathways in LPS-induced RAW 2647 macrophages. Int. J. Mol. Sci..

[18] Deng H (2023). Network pharmacology and experimental validation to identify the potential mechanism of *Hedyotis*
*diffusa* Willd against rheumatoid arthritis. Sci. Rep..

[19] Wang X, Wang Z-Y, Zheng J-H, Li S (2021). TCM network pharmacology: A new trend towards combining computational, experimental and clinical approaches. Chin. J. Nat. Med..

[20] Cho Y-R, Hu X (2022). Network-based approaches in bioinformatics and biomedicine. Methods.

[21] Jiang H, Liu J, Fan C, Wang J, Li W (2021). lncRNAS56464.1 as a ceRNA promotes the proliferation of fibroblast-like synoviocytes in experimental arthritis via the Wnt signaling pathway and sponges miR-152-3p. Int. J. Mol. Med..

[22] Sun Y (2023). LncRNA OIP5-AS1/miR-410-3p/Wnt7b axis promotes the proliferation of rheumatoid arthritis fibroblast-like synoviocytes via regulating the Wnt/β-catenin pathway. Autoimmunity.

[23] Fu Q, Song M-J, Fang J (2022). LncRNA OSER1-AS1 regulates the inflammation and apoptosis of rheumatoid arthritis fibroblast like synoviocytes via regulating miR-1298-5p/E2F1 axis. Bioengineered.

[24] Xu L (2022). The anti-inflammatory effects of *Hedyotis*
*diffusa* Willd on SLE with STAT3 as a key target. J. Ethnopharmacol..

[25] Du H (2019). A novel phytochemical, DIM, inhibits proliferation, migration, invasion and TNF-α induced inflammatory cytokine production of synovial fibroblasts from rheumatoid arthritis patients by targeting MAPK and AKT/mTOR signal pathway. Front. Immunol..

[26] Ardi VC (2009). Neutrophil MMP-9 proenzyme, unencumbered by TIMP-1, undergoes efficient activation in vivo and catalytically induces angiogenesis via a basic fibroblast growth factor (FGF-2)/FGFR-2 pathway. J. Biol. Chem..

[27] Kessenbrock K, Plaks V, Werb Z (2010). Matrix metalloproteinases: regulators of the tumor microenvironment. Cell.

[28] Shi W (2021). METTL3 promotes activation and inflammation of FLSs through the NF-κB signaling pathway in rheumatoid arthritis. Front. Med..

[29] Wei X (2022). Systemic pharmacological verification of Baixianfeng decoction regulating TNF-PI3K-Akt-NF-κB pathway in treating rheumatoid arthritis. Bioorganic Chem..

[30] Wang Y (2023). Pathway and network analyses identify growth factor signaling and MMP9 as potential mediators of mitochondrial dysfunction in severe COVID-19. Int. J. Mol. Sci..

[31] Grillet B (2021). Proteoform analysis of matrix metalloproteinase-9/gelatinase B and discovery of its citrullination in rheumatoid arthritis synovial fluids. Front. Immunol..

[32] Xue M (2014). Endogenous MMP-9 and not MMP-2 promotes rheumatoid synovial fibroblast survival, inflammation and cartilage degradation. Rheumatology (Oxford).

[33] Stojanovic S (2017). Association of tumor necrosis factor-α (G-308A) genetic variant with matrix metalloproteinase-9 activity and joint destruction in early rheumatoid arthritis. Clin. Rheumatol..

[34] Yang Y-X (2023). Bioinformatics analysis of ceRNA regulatory network of baicalin in alleviating pathological joint alterations in CIA rats. Eur. J. Pharmacol..

[35] Wen J-T (2022). Triptolide inhibits cell growth and inflammatory response of fibroblast-like synoviocytes by modulating hsa-circ-0003353/microRNA-31-5p/CDK1 axis in rheumatoid arthritis. Int. Immunopharmacol..

[36] Wang Q-S (2022). Mir204 and Mir211 suppress synovial inflammation and proliferation in rheumatoid arthritis by targeting Ssrp1. Elife.

[37] Xiao J, Wang R, Zhou W, Cai X, Ye Z (2021). LncRNA NEAT1 regulates the proliferation and production of the inflammatory cytokines in rheumatoid arthritis fibroblast-like synoviocytes by targeting miR-204-5p. Hum. Cell.

[38] Zeng S, Tu M (2022). The lncRNA MIAT/miR-181a-5p axis regulates osteopontin (OPN)-mediated proliferation and apoptosis of human chondrocytes in osteoarthritis. J. Mol. Histol..

[39] Wang Z (2021). LncRNA MIAT downregulates IL-1β, TNF-ɑ to suppress macrophage inflammation but is suppressed by ATP-induced NLRP3 inflammasome activation. Cell Cycle..

[40] Deng W (2020). Long noncoding MIAT acting as a ceRNA to sponge microRNA-204-5p to participate in cerebral microvascular endothelial cell injury after cerebral ischemia through regulating HMGB1. J. Cell. Physiol..

[41] Wang N, Yuan Y, Sun S, Liu G (2020). microRNA-204-5p participates in atherosclerosis via targeting MMP-9. Open Med. (Wars).

[42] Farhana A (2023). Gold nanoparticles inhibit PMA-induced MMP-9 expression via microRNA-204-5p upregulation and deactivation of NF-κBp65 in breast cancer cells. Biology (Basel).

[43] Ru J (2014). TCMSP: a database of systems pharmacology for drug discovery from herbal medicines. J Cheminform..

[44] Liu H, Wang J, Zhou W, Wang Y, Yang L (2013). Systems approaches and polypharmacology for drug discovery from herbal medicines: An example using licorice. J. Ethnopharmacol..

[45] Daina A, Michielin O, Zoete V (2019). SwissTargetPrediction: Updated data and new features for efficient prediction of protein targets of small molecules. Nucleic Acids Res..

[46] Shannon P (2003). Cytoscape: A software environment for integrated models of biomolecular interaction networks. Genome Res..

[47] Yu G, Wang L-G, Han Y, He Q-Y (2012). clusterProfiler: An R package for comparing biological themes among gene clusters. OMICS.

[48] Kanehisa M, Goto S (2000). KEGG: Kyoto encyclopedia of genes and genomes. Nucleic Acids Res..

[49] Szklarczyk D (2017). The STRING database in 2017: Quality-controlled protein-protein association networks, made broadly accessible. Nucleic Acids Res..

[50] Hsin KY, Ghosh S, Kitano H (2013). Combining machine learning systems and multiple docking simulation packages to improve docking prediction reliability for network pharmacology. PLoS ONE.

