# Supplementary material for: Identification of Hedyotis diffusa Willd-specific mRNA–miRNA–lncRNA network in rheumatoid arthritis based on network pharmacology, bioinformatics analysis, and experimental verification
Source: Sci Rep. 2024 Mar 15;14:6291. doi: 10.1038/s41598-024-56880-y (PMC10943027; doi:10.1038/s41598-024-56880-y)
Supplement: Supplementary file 1 — Supplementary Information. [file 41598_2024_56880_MOESM1_ESM.docx]

**Supplement Table 1.** The active components and potential targets of HDW.

| PubChem CID | Active Components | Targets |
| --- | --- | --- |
| 5281330 | poriferasterol | PGR, NR3C2 |
| 10514946 | 2-methoxy-3-methyl-9,10-anthraquinone | PTGS1, DRD1, CHRM3, CHRM1, ESR1, SCN5A, CHRM5, PTGS2, CHRM4, RXRA, OPRD1, PDE3A, HRH1, HTR2A, SLC6A2, ADRA1A, HTR2A, CHRM2, ADRA1B, SLC6A3, ADRB2, ADRA1D, SLC6A4, OPRM1, GABRA1, HSP90, PIK3CG, CHRNA7, NCOA2, PKIA |
| 5280794 | stigmasterol | PGR, NR3C2, NCOA2, ADH1C, RXRA, NCOA1, PTGS1, PTGS2, ADRA2A, SLC6A2, SLC6A3, ADRB2, PLAU, LTA4H, MOB, MOA, CTRB1, CHRM3, CHRM1, ADRB1, SCN5A, ADRA1A, CHRM2, ADRA1B |
| 222284 | β-sitosterol | PGR, NCOA2, PTGS1, PTGS2, KCNH2, CHRM3, CHRM1, SCN5A, CHRM4, PDE3A, ADRA1A, CHRM2, ADRA1B, ADRB2, CHRNA2, SLC6A4, OPRM1, BCL2, BAX, CASP9, CASP3, CASP8, PRKCA, TGFB1, PON1, MAP2 |
| 5280343 | quercetin | PTGS1, AR, PPARG, PTGS2, HSP90, NCOA2, TOP2, KCNH2, SCN5A, ADRB2, MMP3, F7, RXRA, ACHE, RELA, EGFR, AKT1, VEGFA, CCND1, BCL2, BCL2L1, CDKN1A, BAX, CASP9, PLAU, MMP2, MMP9, MAPK1, IL10, EGF, RB1, TNF, IL6, AHSA1, CASP3, TP53, ELK1, NFKBIA, POR, ODC1, XDH, CASP8, TOP1, RAF1, PRKCA, MMP1, HIF1A, STAT1, RUNX1T1, ERBB2, PPARG, ACACA, HMOX1, CYP3A4, CAV1, MYC, F3，GJA1, CYP1A1, ICAM1, IL1B, CCL2, SELE, VCAM1, PTGER3, PRKCB, BIRC5, DUOX2, NOS3, HSPB1, IL2, NR1I2, CYP1B1, CCNB1, PLAT, THBD, SERPINE1, IFNG, IL1A, MPO, TOP2A, NCF1, HAS2, GSTP1, NFE2L2, AHR, PSMD3, SLC2A4, CXCL11, CXCL2, DCAF5, NR1I3, CHEK2, INSR, CLDN4, PPARA, PPARD, HSF1, CRP, CXCL10, CHUK, SPP1, RUNX2, RASSF1, E2F1, E2F2, ACP3, CTSD, IGFBP3, IGF2, CD40LG, IRF1, ERBB3, PON1,DIO1, PCOLCE, NPEPPS, HK2, NKX3-1, RASA1, GSTM1, GSTM2 |
| 5280863 | kaempferol | NOX4, AKR1B1, XDH, TYR, FLT3, CA2, ALOX5, CA7, HSD17B2, ABCC1, HSD17B1, AHR, CA12, ESRRA, ABCB1, CYP1B1, ABCG2 |
| 5280460 | scopoletin | CA7, CA12, CA9 |
| 637542 | p-Coumaric acid | AKR1B1, CA1, CA2, CA3, CA4, CA9, CA5A, CA5B, CA6, CA7, CA12, CA14, ESR2 |
| 72 | 3，4-Dihydroxybenzoic acid | CA2, CA7, CA1, CA6, CA12, CA14, CA9, CA4 |
| 445858 | Ferulic acid | CA2, CA7, CA1, CA6, CA12, CA14, CA9, CA5A, |
| 135 | p-Hydroxybenzoic acid | CA2, CA7, CA1, CA3, CA6, CA12, CA14, CA9, CA4, CA5B, CA5A, CA13 |

**Supplement Table Table 2.** The list of genes contributing to the the 20 selected pathways.

| ID | Description | Gene |
| --- | --- | --- |
| hsa04657 | IL-17 signaling pathway | PTGS2/MMP9/MMP3/MMP1/IFNG/CXCL2/CXCL10/CCL2/CASP8 |
| hsa04668 | TNF signaling pathway | PTGS2/MMP9/MMP3/IRF1/CXCL2/CXCL10/CCL2/CASP8 |
| hsa05417 | Lipid and atherosclerosis | NCF1/MMP9/MMP3/MMP1/CYP1A1/CXCL2/CCL2/CASP8 |
| hsa05215 | Prostate cancer | PLAU/MMP9/MMP3/EGFR/EGF/CDKN1A/AR |
| hsa05163 | Human cytomegalovirus infection | PTGS2/PTGER3/PRKCB/EGFR/CDKN1A/CCL2/CASP8 |
| hsa05165 | Human papillomavirus infection | SPP1/PTGS2/IRF1/EGFR/EGF/CDKN1A/CASP8 |
| hsa05219 | Bladder cancer | MMP9/MMP2/MMP1/EGFR/EGF/CDKN1A |
| hsa05160 | Hepatitis C | IFNG/EGFR/EGF/CXCL10/CDKN1A/CASP8 |
| hsa04062 | Chemokine signaling pathway | PRKCB/PIK3CG/NCF1/CXCL2/CXCL10/CCL2 |
| hsa05205 | Proteoglycans in cancer | PRKCB/PLAU/MMP9/MMP2/EGFR/CDKN1A |
| hsa05171 | Coronavirus disease - COVID-19 | PRKCB/MMP3/MMP1/EGFR/CXCL10/CCL2 |
| hsa05206 | MicroRNAs in cancer | PTGS2/PRKCB/PLAU/MMP9/EGFR/CDKN1A |
| hsa05323 | Rheumatoid arthritis | MMP3/MMP1/IFNG/CXCL2/CCL2 |
| hsa04066 | HIF-1 signaling pathway | PRKCB/IFNG/EGFR/EGF/CDKN1A |
| hsa05418 | Fluid shear stress and atherosclerosis | NCF1/MMP9/MMP2/IFNG/CCL2 |
| hsa04921 | Oxytocin signaling pathway | PTGS2/PRKCB/PIK3CG/EGFR/CDKN1A |
| hsa05164 | Influenza A | PRKCB/IFNG/CXCL10/CCL2/CASP8 |
| hsa05167 | Kaposi sarcoma-associated herpesvirus infection | PTGS2/PIK3CG/CXCL2/CDKN1A/CASP8 |
| hsa05415 | Diabetic cardiomyopathy | PRKCB/NCF1/MMP9/MMP2/CTSD |
| hsa05207 | Chemical carcinogenesis - receptor activation | PRKCB/EGFR/EGF/CYP1A1/AR |


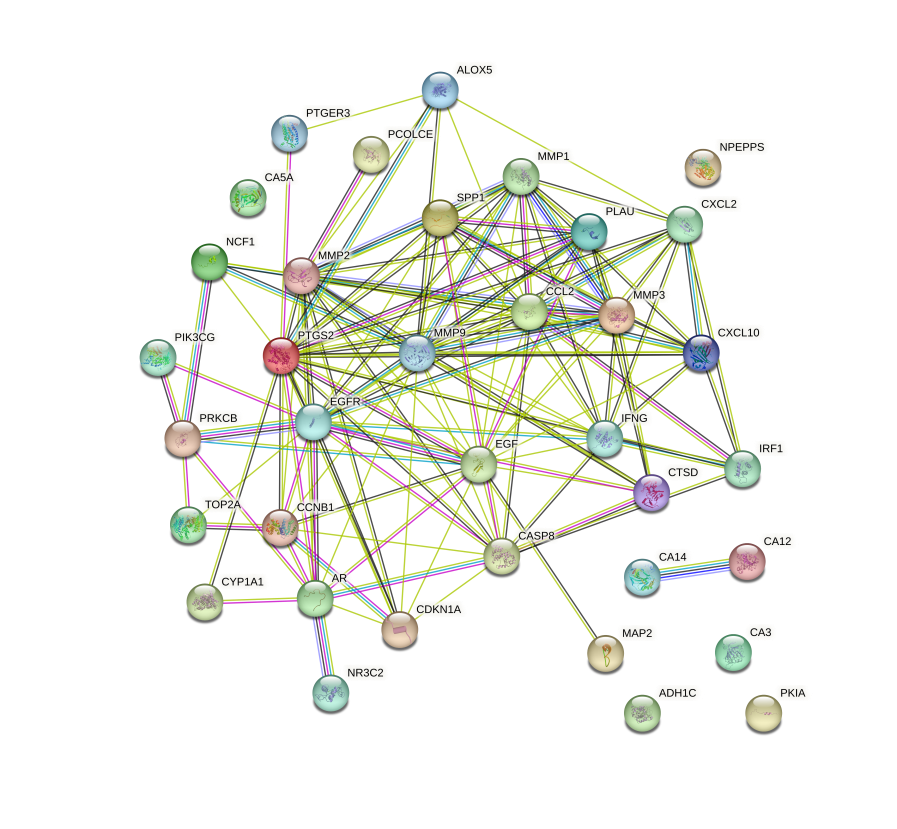


**Supplement Figure 1.** The PPI network of 36 common targets.

**Supplement Table 3.** Key targets of HDW against RA.

| Uniprot ID | Gene name | Protein name | DC | BC | CC | NC |
| --- | --- | --- | --- | --- | --- | --- |
| P35354 | PTGS2 | Prostaglandin G/H synthase 2 | 21 | 126.81126 | 0.8 | 19.578594 |
| P01133 | EGF | Pro-epidermal growth factor | 19 | 93.98236 | 0.7567568 | 15.824019 |
| P14780 | MMP9 | Matrix metalloproteinase 9 | 19 | 41.78586 | 0.7567568 | 17.281862 |
| P08253 | MMP2 | Matrix Metalloproteinase 2 | 19 | 89.5254 | 0.7567568 | 15.65762 |
| P13387 | EGFR | Epidermal growth factor receptor | 18 | 98.87987 | 0.7368421 | 15.788057 |
| P13500 | CCL2 | C-C motif chemokine 2 | 16 | 22.94412 | 0.68292683 | 14.288889 |
| P08254 | MMP3 | Matrix Metalloproteinase 3 | 13 | 5.861977 | 0.6363636 | 11.836868 |
| P01579 | IFNG | Interferon gamma | 13 | 7.6516595 | 0.6363636 | 11.623737 |
| Q14790 | CASP8 | Caspase-8 | 13 | 17.64127 | 0.6511628 | 10.394697 |
| P03956 | MMP1 | Matrix Metallopeptidase 1 | 12 | 2.7826118 | 0.62222224 | 11.112122 |
| P10451 | SPP1 | Secreted Phosphoprotein 1 | 12 | 2.0095239 | 0.62222224 | 10.9616165 |
| P02778 | CXCL10 | C-X-C Motif Chemokine Ligand 10 | 11 | 3.8373015 | 0.5833333 | 9.888889 |
| P10275 | AR | Androgen Receptor | 11 | 73.36072 | 0.62222224 | 7.775 |
| P00749 | PLAU | Plasminogen Activator, Urokinase | 10 | 1.5793651 | 0.59574467 | 9.388889 |
| P19875 | CXCL2 | C-X-C Motif Chemokine Ligand 2 | 10 | 5.134524 | 0.56 | 8.4 |
| P14635 | CCNB1 | G2/mitotic-specific cyclin-B1 | 9 | 13.107359 | 0.59574467 | 7.75 |

**Supplement Table 4.** Specific results of LASSO, SVM-RFE and RF.

| Algorithm | Gene name |
| --- | --- |
| LASSO | PTGS2/MMP9/EGF/MMP2/SPP1/CXCL10 |
| SVM | MMP9 |
| RF | MMP9/CCL2/PTGS2 |

**
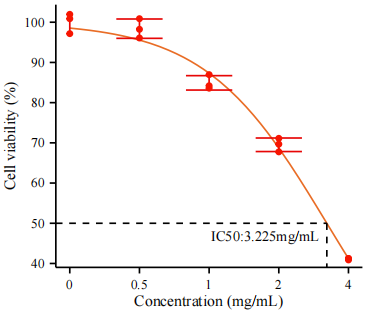
**

**Supplement Figure 2.** IC50 experiment of RA-FLS cells treated with HDW for 48 hours.

MMP9

MMP9

MMP9


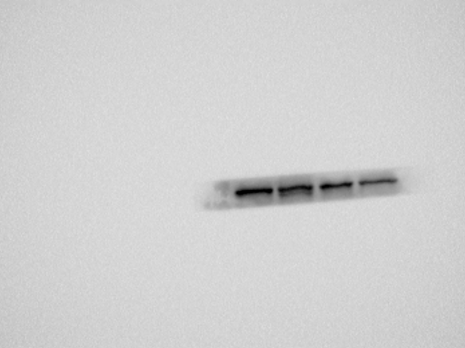

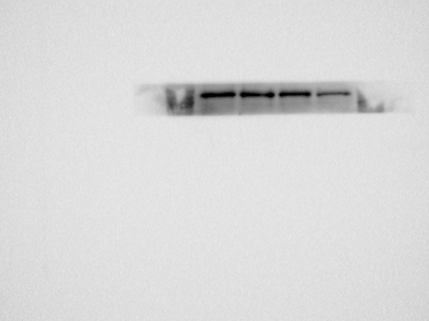

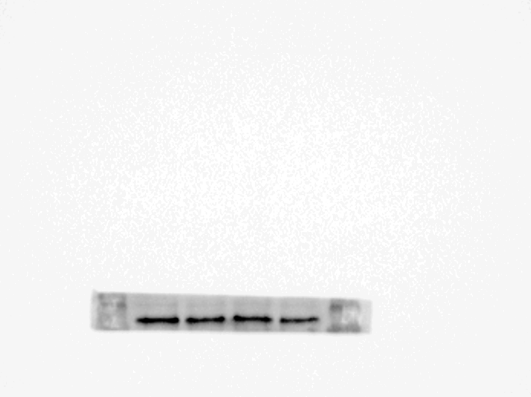


β-actin

β-actin

β-actin

**
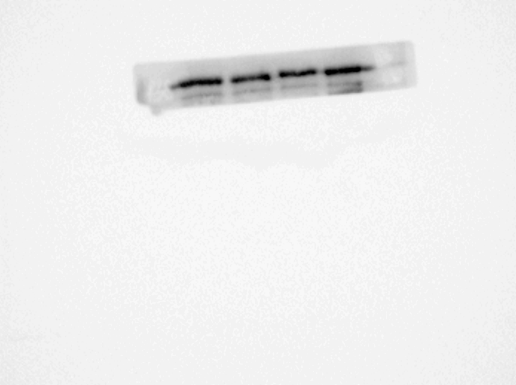

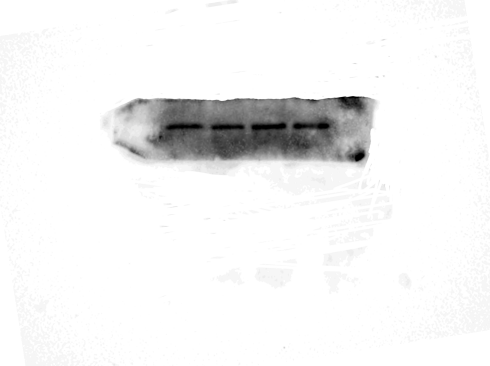

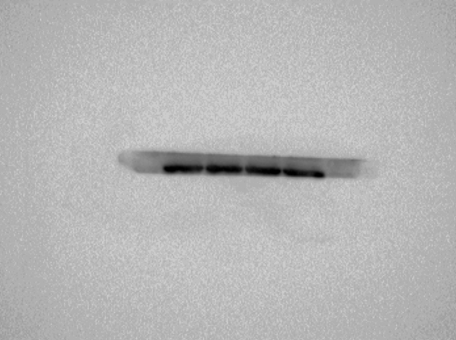
**

**Supplement Figure 3.** The original immunoblot images of MMP9 and β-actin. The experiment was independently repeated three times.


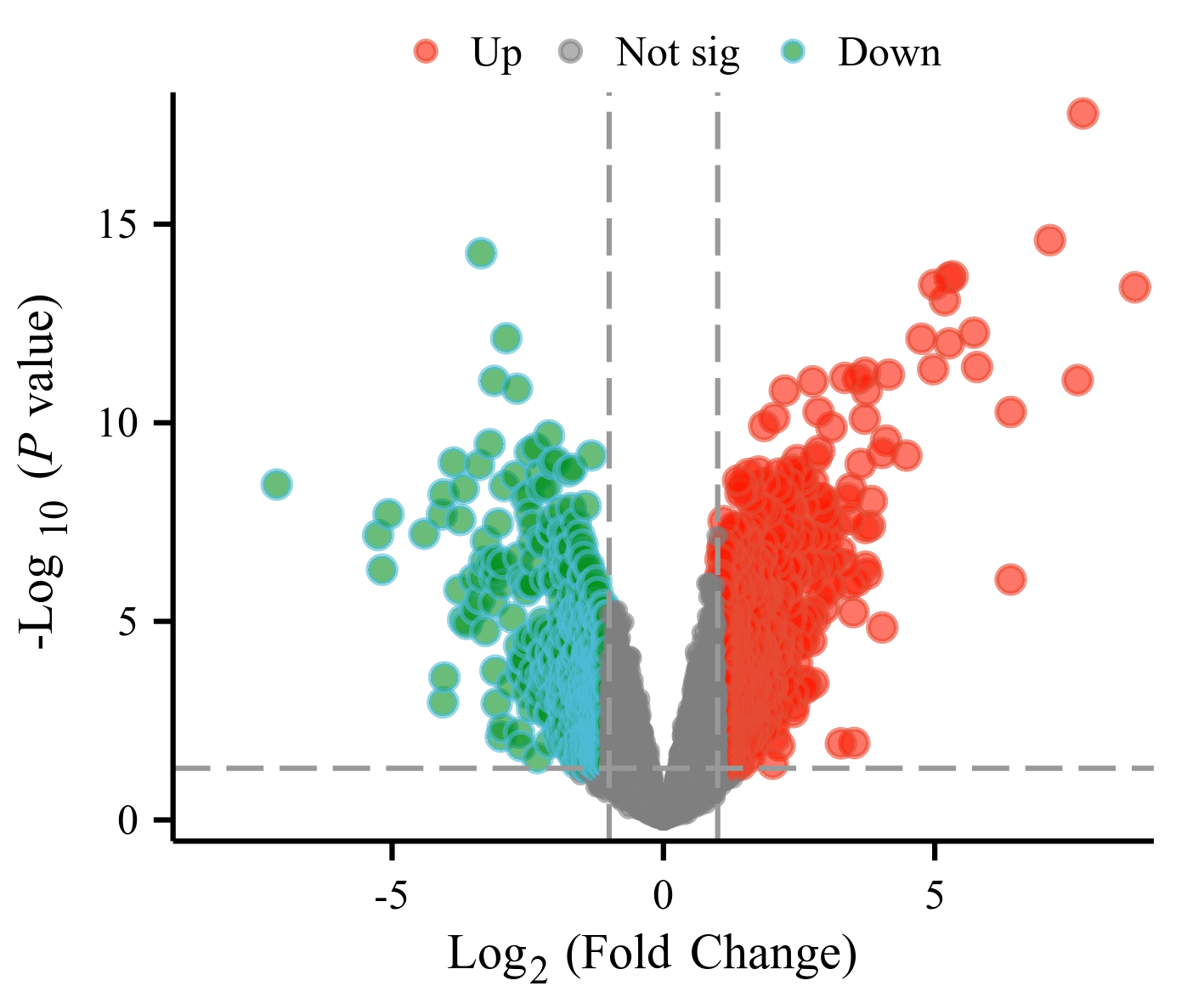

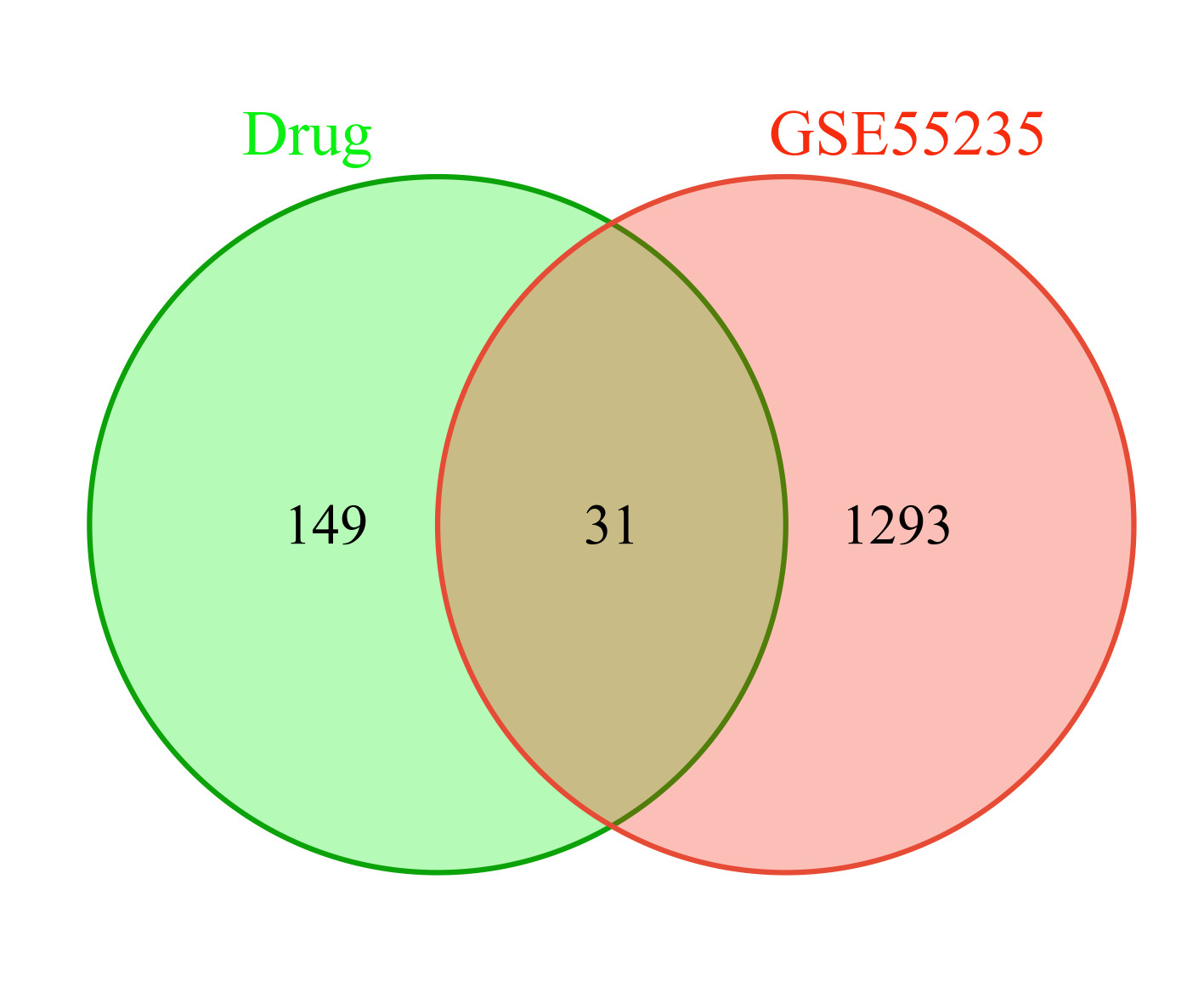

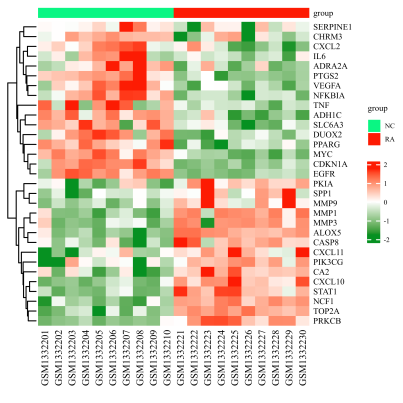


**Supplement Figure 4**. HDW-regulated differentially expressed genes identified in the datasets GSE55235.


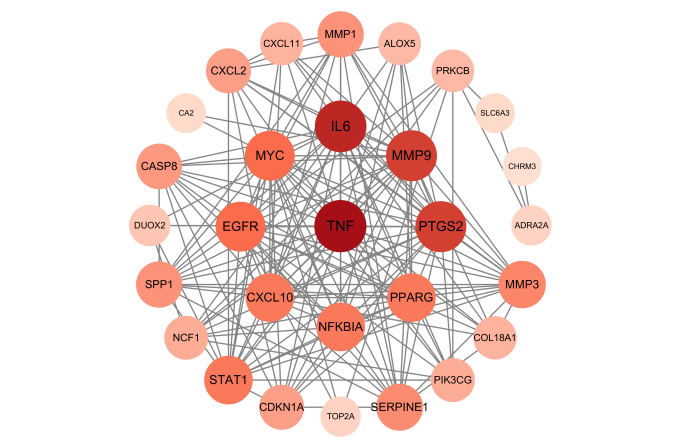

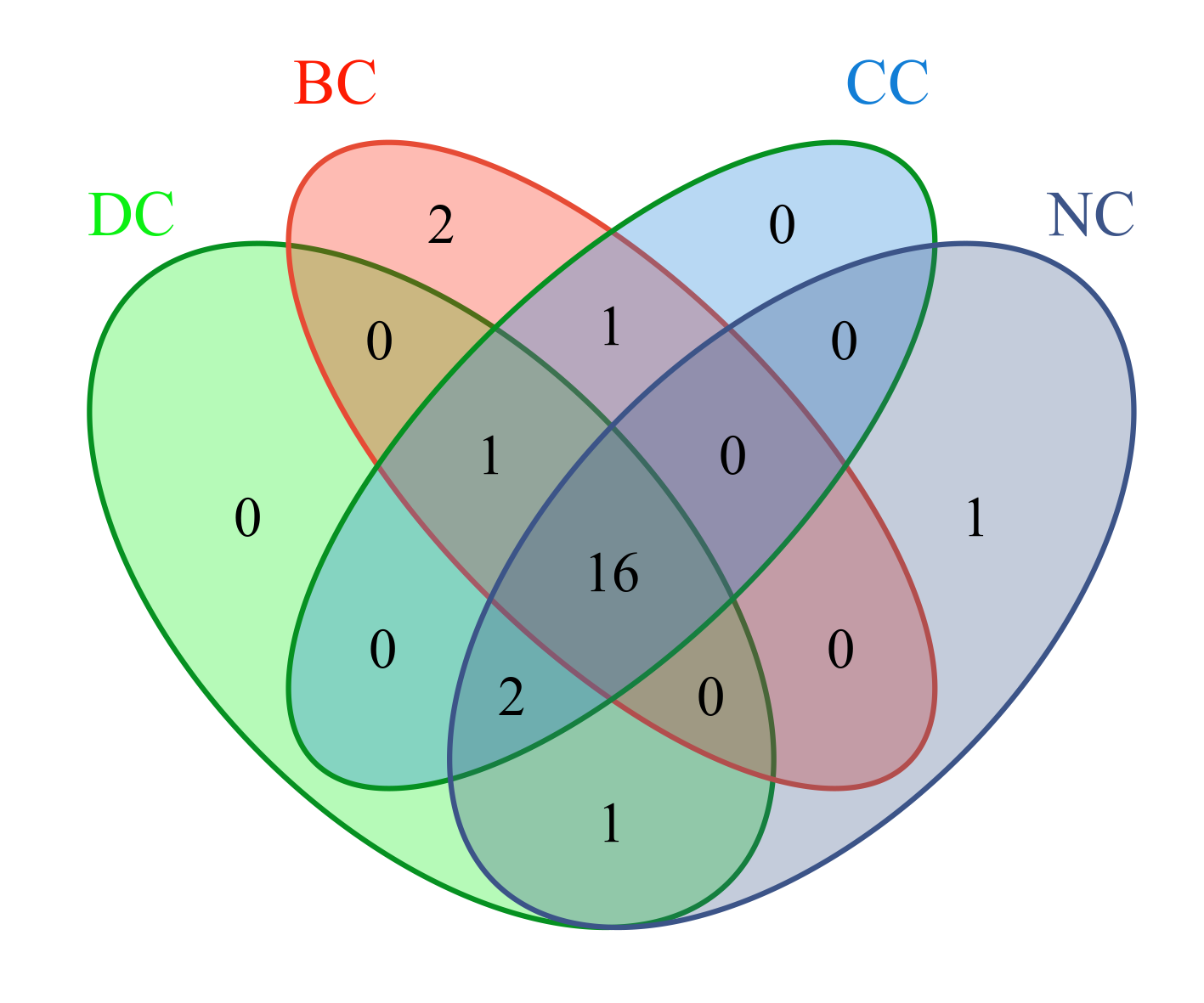

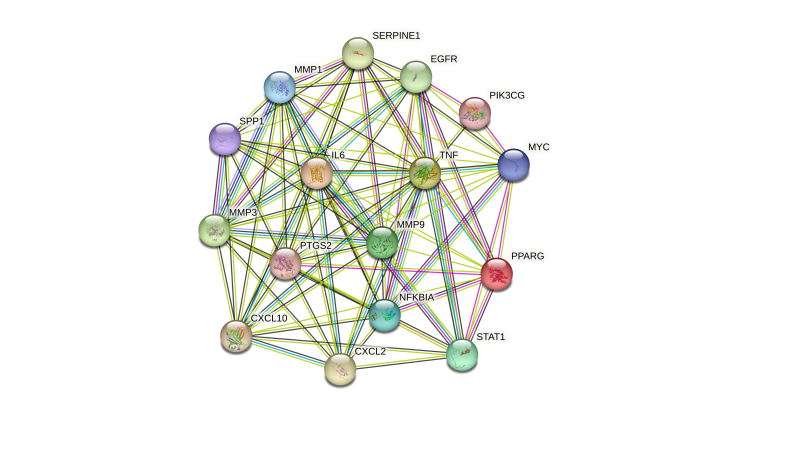


**Supplement Figure 5**. Screening hub targets through PPI network.


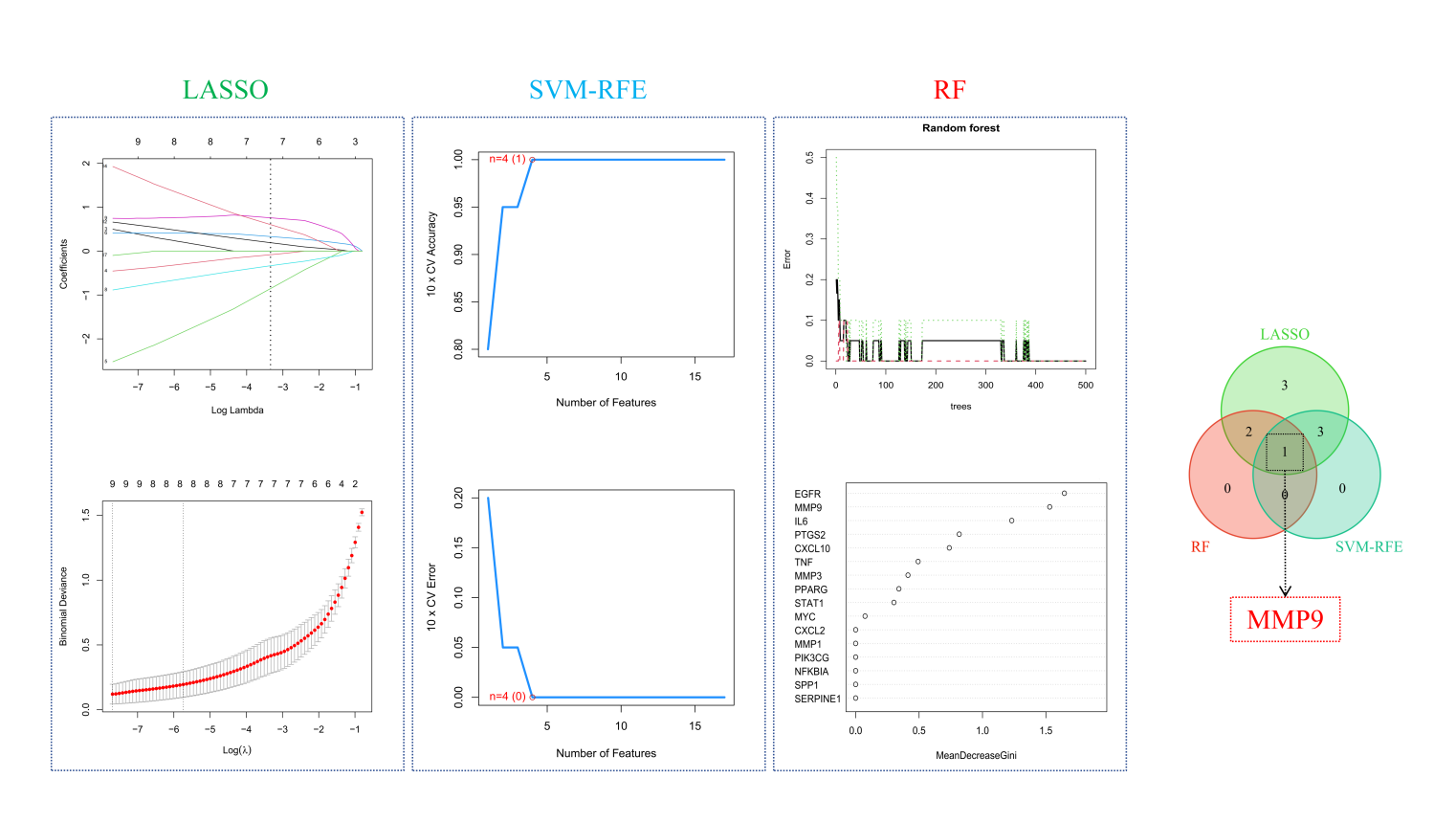


**Supplement Figure 6**. MMP9 was identified as the core gene of RA through three computational learning algorithms.

**Supplement Table 5.** Primer sequences for all genes.

| Gene Symbol | NCBI ID |  | Sequences (5’-3’) |
| --- | --- | --- | --- |
| MMP9 | [4318](https://www.ncbi.nlm.nih.gov/gene/4318) | F | TGTACCGCTATGGTTACACTCG |
|  |  | R | GGCAGGGACAGTTGCTTCT |
| miR-204-5p | [406987](https://www.ncbi.nlm.nih.gov/gene/406987) | RT | CGTTCCCTTTGTCATCCTATGCCT |
|  |  | F | CGTTCCCTTTGTCATCCTATGCCT |
|  |  | R | ATCCAGTGCAGGGTCCGAGG |
| MIAT | [440823](https://www.ncbi.nlm.nih.gov/gene/440823) | F | TTTCTGCCTGTTTGCTGCTT |
|  |  | R | CCTCCCTACTGGGTCTCCTT |
| U6 | [26827](https://www.ncbi.nlm.nih.gov/gene/26827) | RT | AACGCTTCACGAATTTGCGT |
|  |  | F | CTCGCTTCGGCAGCACA |
|  |  | R | AACGCTTCACGAATTTGCGT |
| β-actin | [60](https://www.ncbi.nlm.nih.gov/gene/60) | F | ACCCAGAAGACTGTGGATGGC |
|  |  | R | TCAGATCCACGACGGACACAT |
